# Supplementary material for: The magnitude of, and factors associated with, loss to follow-up among patients treated for sexually transmitted infections: a multilevel analysis
Source: BMJ Open. 2017 Jul 17;7(7):e016864. doi: 10.1136/bmjopen-2017-016864 (PMC5726144; doi:10.1136/bmjopen-2017-016864)
Supplement: Author's manuscript [file bmjopen-2017-016864.draft_revisions.pdf]

BMJ Open

The magnitude and factors associated with loss to follow-up among patients treated for sexually transmitted infections: A multilevel analysis

Journal:	*BMJ Open*
Manuscript ID	bmjopen-2017-016864
Article Type:	Research
Date Submitted by the Author:	15-Mar-2017

Complete List of Authors:	Tsadik, Mache; Mekelle University<br>Berhane, Yemane ; Addis Continental Institute of Public Health<br>Worku, Alemayehu; Addis Ababa University<br>Terefe, Wondwossen; Mekelle University

**Primary Subject Heading**:	Infectious diseases
Secondary Subject Heading:	Public health, Sexual health, Epidemiology
Keywords:	PUBLIC HEALTH, Infection control < INFECTIOUS DISEASES, Epidemiology < INFECTIOUS DISEASES

SCHOLARONE™
Manuscripts

For peer review only

The magnitude and factors associated with loss to follow-up among patients treated for sexually transmitted infections: A multilevel analysis

Mache Tsadik [1*,] Yemane Berhane[2], Alemayehu Worku[3], Wondwossen Terefe[1]

Mache Tsadik (adhana2008@gmail.com)

Yemane Berhane (yemaneberhane@gmail.com)

Alemayehu Worku (alemayehuwy@yahoo.com)

Wondwossen Terefe (wlerebo@uwc.ac.za)

Affiliation

1 College of health science, Mekelle University, Tigray, Ethiopia

2 Addis Continental Institute of Public Health, Addis Ababa, Ethiopia

3 Addis Ababa University, Addis Ababa, Ethiopia

* Corresponding Author, adhana2008@gmail.com

For peer review only

For peer review only - http://bmjopen.bmj.com/site/about/guidelines.xhtml

Abstract

Objectives: Research on loss to follow up among patients attending STI care in Sub-Saharan Africa was rarely documented. The objective of this study was to investigate individual- and facility-level factors associated with lost to follow-up (LTFU) among patients treated for sexually transmitted infections (STIs) in Ethiopia.

Methods: A prospective cohort study was conducted among patients attending care for STIs in selected facilities from January to June 2015 in Tigray region of Ethiopia. LTFU was ascertained if a patient didn't present in person to the same facility within 7-days of the initial contact. Multilevel logistic regression was used to identify factors associated with LTFU.

Results: Out of 1082 patients, 59.80% (n=647) were LTFU. The individual-level factors associated with LTFU included: having multiple partners (AOR=2.89, 95%CI: 1.74-4.80), being male (AOR=2.23, 95%CI: 1.63-3.04), having poor knowledge of STI transmission (AOR=2.08, 95%CI: 1.53-2.82), having college level education (AOR=0.38, 95%CI: 0.22-0.65) and low perceived stigma (AOR=0.60, 95% CI: 0.43-0.82). High patient flow (AOR=3.06, 95%CI: 1.30-7.18) and medium health index score (AOR=2.80, 95%CI: 1.28-6.13) were facility-level factors associated with LTFU.

Conclusions: To improve patients retention in STI follow up care in Ethiopia, focused interventions targeting those who were more likely to be LTFU, particularly patients with multiple partners, male index cases and patients attended facilities with high patient flow, might be paramount.

Key words: sexually transmitted infections, partner notification, loss to follow-up, multilevel analysis

For peer review only

For peer review only - http://bmjopen.bmj.com/site/about/guidelines.xhtml

Strengths and limitations of this study

- Since this study is among the few, it helps to guide the clinical and public health practice in the management of STIs particularly during the follow up care.
- Baseline information was given to study participants to return for follow up and inform sexual partners within a week period.
- Advanced analytic method was used that allow simultaneous examination of individual and facility level factors affecting follow up care among patients attending STI care
- This study failed to address reasons for LTFU because there was no established participant tracing mechanism for patients who were LTFU in the context of this study.
- Extrapolation of results beyond the study participants should be made with caution because substantial number of patients also seeks care from private facilities.

For peer review only

For peer review only - http://bmjopen.bmj.com/site/about/guidelines.xhtml

Introduction:

Disclosure of the diagnosis of sexually transmitted infections (STIs) to partners is an important preventive strategy. [1,2] Partner notification (PN) is an effective method of case finding, reducing transmission and preventing complications.[3] Successful implementation of PN also reduces the magnitude of STIs in the community.[4] However, efforts to prevent the transmission of STIs between partners are challenged by failure to notify partner and low return for follow up services.

Early notification of sexual partners has shown a substantial reduction in risk of reinfection and complications following STIs.[5] Reports showed that index cases usually notify their partners within a week [6,7], which is advantageous in preventing STI risks. Delay in PN has adverse effects and is often due to stigma, shame, fear of violence and lack of contact information.[7]

PN is the process of informing, diagnosing, treating and advising on future infections.[8] Follow-up care is mandatory to evaluate PN processes. Ascertaining notification status would be unlikely if index case is LTFU. [9] Therefore it is important to schedule patients for follow-up care to ensure treatment compliance and to assess risk of re-infection.[10] However, studies reported that low return for follow-up care is attributed to the poor quality of care in STI clinics in both developed and developing countries. [11-14]

Several individual factors contribute to LTFU during the follow-up of STI patients. Accordingly, males compared to females [15], single compared to married [14], uneducated/less educated compared to those educated at college level [16], individuals with poor knowledge of STI compared to those with better knowledge[17], and individuals that did not intended to notify partners compared to those intended to notify had shown a greater likelihood of being LTFU. [18]

In Ethiopia, the STI treatment guidelines recommend the syndromic management approach and initiation of treatment at the first contact. However, the guidelines recommend a follow-up care to promote partner notification, assess responses to treatment and provide sexual health education.[19] Despite the public health importance of follow-up care, studies rarely examined the magnitude and factors associated with LTFU among patients treated for STIs using a multilevel level regression model. Therefore, we aimed to investigate individual and facility-level factors affecting LTFU among STI patients attending public health facilities in North Ethiopia.

For peer review only - http://bmjopen.bmj.com/site/about/guidelines.xhtml

Methods:

A prospective cohort study was conducted among patients attending public health facilities for STI care from January to June 2015 in Tigray region of Ethiopia. Public health facilities in Ethiopia provide low cost treatment for common STIs. A primary health care unit in Ethiopia, which includes at least one health center and 5-7 satellite health posts, is supposed to serve 25,000 populations. The primary health care units are highly accessible and provide treatment for common illness and implement preventive health services either for free or at low cost.

Initially, all self referral cases presented with STI syndrome/s in the selected facilities and had sexual intercourse with in three months period prior to the study were enrolled in the study. Then, patients who fulfill the criteria and volunteer to participate in the study were informed to notify potential partners and to return for follow up within a week period. Patients were also instructed about whom to contact including the room number when returned for follow up. In addition, patients were given a card at initial contact with the aim to establish a link with the research assistant during their follow up visit. A minimum of two research assistants were also assigned in a health facility to capture patients returned for follow up within the specified period. Considering these prior information, LTFU was ascertained if the index case didn't present in person to the same health facility within a week period provided that each index case was informed to return for follows up at baseline and classified as "Yes". Patients who were not LTFU were referred to as "in follow up care" and classified as "No" in the context of this study.

The study was conducted in public health facilities that were reporting comparatively high monthly STI patient case load. The private facilities were excluded because of lack of monthly STI record which may introduce selection bias and affect the generalizability of the study. Sample size was primarily calculated to determine predictors of PN for STIs by considering 40% of married individuals who notified partners [20] and odds ratio of 1.5 at 95% CI and power of 80% with a non response rate of 20% which gives a total of 1095. Those who fulfilled the criteria and voluntarily consent to participate in the study (n=1082) were consecutively interviewed at baseline and then appointed to return for follow-up within 7-days.

The study tool was developed by reviewing relevant literatures and then adapting to the context of our study. The questionnaire comprises of sociodemographic, behavioral and psychosocial components. The questionnaire was pre-tested in a similar context. Data were collected by

For peer review only - http://bmjopen.bmj.com/site/about/guidelines.xhtml

trained nurses through face to face interview. Interviews were conducted at the clinic exist after obtaining an informed verbal consent.

The description of variables used in the analysis and their measurement is indicated in table 1. According to the HMIS report of Tigray Regional Health Bureau, health facilities were classified into level using index score as low (<50), medium(50-74.9) and high(≥75).[21] However, among the facilities selected for this study no health facility was under the category of "low" and analysis was made using medium and high category.

Statistical analysis:

Index cases were advised to notify partners and return for follow-up at baseline, the initial contact. Based on this, LTFU was ascertained if the index-case didn't present in person to the same health facility within 7-days. Patients not LTFU were referred as "in follow-up care". One week is taken to define LTFU considering partner notification was most likely to happen within 2-3 days. [7]

In multilevel analyses, a null model (Model-1) with no covariates was used to assess the presence of significant clustering in LTFU. For individual level factors (Model-2), the analysis considered sex, marital status, education, number of partners, types of partnership, knowledge of STI transmission and complication as well as perceived stigma. In Model-3, facility-level variables such as health facility index and STI patient flow were considered for analysis. Finally, Model-4 included both individual and health facility-level variables. The command "xtmelogit" in Stata 12 was used for all model estimations. In the multilevel analyses, the magnitudes of relationship between the dependent variable and each of the independent variables (i.e. fixed effects) were assessed using odds ratios and their confidence intervals. To evaluate the significance of facility-level clustering of the dependent variable (i.e. random effects), log-likelihood ratio tests were employed. We used variable inflation factor (VIF) to check Multi-Collinearity amongst the individual and facility-level factors.

For peer review only - http://bmjopen.bmj.com/site/about/guidelines.xhtml

Results:

In this study, 1082 patients who received STI care in selected health facilities were each followed for a week. Out of those, 647 (59.80% at 95%CI: 56.88-62.72) patients who attended the study facilities did not comeback for follow up care, were LTFU (figure 1). The mean age of the cohort population was 26.4 years (SD=7.6). LTFU was high among males (71%) and illiterates (73.6%). Casual and multiple partners were higher among LTFU cases than on follow-up care. High proportion of LTFU was seen among facilities with high patient flow. However, no difference in LTFU was identified in regard to age and residence. The profile of respondents was indicated in table 2.

Multilevel logistic regression analysis:

After controlling the potential predictors at individual and facility-level, the odds of LTFU were higher among index cases with multiple partners (AOR=2.89, 95%CI: 1.74-4.80), males (AOR=2.23, 95%CI: 1.63, 3.04), poor knowledge of STI transmission (AOR=2.08, 95%CI: 1.53-2.82) and poor knowledge of STI complication (AOR=1.56, 95% CI: 1.15-2.12). This study also revealed that LTFU was less likely among highly educated individuals (AOR=0.38, 95%CI: 0.22-0.65) and perceived low stigma (AOR=0.60, 95%CI: 0.43-0.82). Patients who received care in facilities with high patient flow were three times (AOR=3.06, 95%CI: 1.30-7.18) more likely to LTFU than their counterparties. The likelihood of LTFU was also nearly three times (AOR=2.80, 95%CI: 1.28-6.13) higher among respondents received care in facilities with medium index score compared to highest index score (Table 3).

For peer review only

Discussion:

This study aimed to identify individual and facility-level factors associated with LTFU. While LTFU was more likely among male index cases, those having multiple partners and poor knowledge of STIs LTFU were less likely among those highly educated and expressed low perceived stigma. High patient load and medium index score were found significantly associated with LTFU among facility-level factors.

The magnitude of LTFU in the present study (59.8%) is consistent with other studies.[13 14] However, it is higher than study that reported LTFU of 40%.[12] Early response to treatment, relief of symptoms and fear of stigma to turn back to the same facility for follow up might attribute LTFU to be inflated.[7 22] Once patients are lost to STI care, their compliance to prescribed treatment might be low. This leads not only to serious complications among such individuals but also leads to the development of STI drug resistance and contribute to the ongoing spread of STI in the community.

Our finding showed that males were more likely to LTFU than females.[15] It has been suggested that males were more likely engage in risk sexual behaviors than females.[23] Besides, permissive attitude to male dominance and decision making encourages males to take part in risky sexual matters.[24] As a result, more males LTFU than females may be because of risk related stigma.

The less likelihood of LTFU among educated individuals in this study is consistent to a previous study.[14] This may suggest that education is an important factor to adhere to medical care.[25] Besides, highly educated individuals have higher knowledge of STIs and individuals with high knowledge of STIs are less likely to LTFU.[17] Lack of knowledge of STI complication was associated with having multiple partners [26] which may decrease individual's motivation to attend follow-up care because of stigma.

Perceived stigma was significantly associated with LTFU. As STI linked stigma affects willingness to notify partners [27], those unwilling to notify partners may not return for follow-up because of shame, embracement and provider's expectation.[28] But those who perceive low stigma may be confident enough to notify partners and have the courage to return for follow-up.

LTFU is higher among patients from facilities with high patient flow in the present study. A study conducted among HIV infected patients reported the association of high patient load with

For peer review only - http://bmjopen.bmj.com/site/about/guidelines.xhtml

high proportion of LTFU.[29] High patient load might limit the provider's time to provide adequate care that potentially influences index cases to LTFU. It may also be associated with perceived stigma from clinic attendants that attribute to LTFU. The odds of LTFU were also lower among patients attending facilities with high index score in our study. Similarly, patient focused study conducted in Nigeria has shown that patients with high quality score were less likely to LTFU.[30] This may indicate that high quality care motivates patients to be retained in follow-up care.

This study has some important limitations. First, reasons for LTFU were not documented as this study didn't assume participant tracing mechanism for those patients who did not come back to the health facilities within the time frame. As a result, these patients were regarded as LTFU while patients could possibly notify partners and engaged in follow up care at another facility because of STI linked stigma. This may attribute LTFU to be overestimated in this study. Second, considerable differences in magnitude of LTFU might occur because of lack of uniform definition on LTFU, thus comparism should be made with caution. Third, study participants who returned for follow-up after the scheduled time were not captured. Fourth, since the study was conducted among self referred patients from public health facilities, extrapolation of results beyond the study participants should be made with caution. Behavioral information was all self-reported which may pose reliability issue because of social desirability and recall bias.

Despite the stated limitations, this study is one of the few studies ever conducted on LTFU among patients treated for STIs which helps to guide practitioners for focused intervention. Informing patients to notify partners and to return for follow up at baseline is also strength of this study which enhances patients return though high to LTFU was observed. This study also employed advanced method of analysis that allows the simultaneous analysis of individual and facility-level factors.

Conclusion:

Overall, the magnitude of LTFU is very high in North Ethiopia. This implies that many partners remained untreated for STIs which challenges the STI control strategy. Our study found that those who were engaged in multiple partnership and male index cases were more likely to be LTFU among patients treated for STIs. The likelihood of LTFU was also more likely among STI patients attending facilities with high patient flow. These findings will help clinicians and public

For peer review only

For peer review only - http://bmjopen.bmj.com/site/about/guidelines.xhtml

health practitioners to recognize patients that require additional support to remain in care such as provision of intensive adherence counseling and adequate information. The need for standardized follow up care and cost effective tracing mechanism is important to retain and trace patients who are at risk of LTFU.

Acknowledgement

We would like to acknowledge Mekelle University and Addis Continental Institute of Public Health for the overall support. This research was partially funded by African Doctoral Dissertation Research Fellowship award (ADDRF) (Grant Number: ADF 015). We would also like to thank Tigray Health Bureau and Directors of the selected health facilities for facilitating the conduct of this study. We are also grateful to all study participants, data collectors and supervisors without whom this work would not have been possible.

Authors' contribution

The study was designed by MT, YB, AW and WT. MT was responsible for data collection, analysis and drafting the manuscript. YB revised the study design and the manuscript. AW supervised the data collection and analysis, and revising the manuscript, and contributed to interpretation of the analysis. WT participated in the analysis and interpretation of the data, as well as revised the manuscript. All authors have read and approved the final manuscript.

Funding: MT is funded by Mekelle University and African Population Health Research Center (APHRC). The funders had no role in the gathering or analysis of the data and no role in the writing of the manuscript or the decision to submit for publication.

Competing interests: The authors declare that they have no competing interests.

Ethical Approval: Mekelle University Health Research Ethical Review Committee (HRERC) gave ethical approval. ERC 0458/2014, date approved: 01 November 2014. The study participants gave informed consent before being involved in the study.

Data sharing statement: No additional data available

For peer review only - http://bmjopen.bmj.com/site/about/guidelines.xhtml

Reference:

1. WHO. Global strategy for the prevention and control of sexually transmitted infections: 2006-2015. Geneva: WHO, 2007.

2. Tiplica GS, Radcliffe K, Evans C et al. European guidelines for the management of partners of persons with sexually transmitted infections, 2015.

3. Taylor D. Outcomes Related to STI Partner Notification. October, 2013.

4. Ward H, Bell G. Partner notification. *Medicine* 2014;42(6):314-17.

5. Low N, Heijne JC, Herzog SA and Althaus CL Reinfection by untreated partners of people treated for Chlamydia trachomatis and Neisseria gonorrhoeae: mathematical modelling study. *Sex Transm Infect.* 2014;90(3):254-6.

6. Tafuma TA, Ntwayagae BC, Moalafhi CK et al. Patient-initiated sexual partner notification in Botswana and time taken for sexual contacts to report for treatment. *S Afr Med J* 2014;104(1):42-44.

7. CDC. Sexually Transmitted Diseases Treatment Guidelines, 2015.

8. Azariah S. Partner notification for sexually transmitted infections. Why can't we talk about it? *Journal of the New Zealand Medical Association* 2012;125(1355).

9. Low N, Heijne JC, Herzog SA, et al. Reinfection by untreated partners of people treated for Chlamydia trachomatis and Neisseria gonorrhoeae: mathematical modelling study. *Sex Transm Infect* 2014;90(3):254-6.

10. Lazaro N. Sexually Transmitted Infections in Primary Care 2013 (RCGP/BASHH). available at www.rcgp.org and www.bashh.org/guidelines, 2013.

11. Malik AI, Huppert JS. Interval to treatment of sexually transmitted infections in adolescent females. *J Pediatr Adolesc Gynecol* 2007;20(5):275-79.

12. Huppert J, Reed JL, Munafo JK, et al. Improving Notification of Sexually Transmitted Infections: A Quality Improvement Project and Planned Experiment. *PEDIATRICS* 2012;130 (2):415-22.

13. Buchsbaum A, Gallo MF, Whiteman MK, et al. Sexually Transmitted Disease Partner Notification among African-American, Adolescent Women. *Infectious Diseases in Obstetrics and Gynecology;* 2014;Article ID 619632 9 pages.

14. Tang W, Huan X, Zhang Y et al. . Factors Associated with Loss-toFollow-Up during Behavioral Interventions and HIV Testing Cohort among Men Who Have Sex with Men in Nanjing, China. *PLoS ONE* 2015;10(1):e115691.

For peer review only - http://bmjopen.bmj.com/site/about/guidelines.xhtml

15. Burnet Institute. Partner notification of sexually transmitted infections in New South Wales: An informed literature review, 2010. , 2014.

16. Ahmed I, Gugsa ST, Lemma S, et al. Predictors of loss to follow-up before HIV treatment initiation in Northwest Ethiopia: a case control study. *BMC Public Health* 2013;13(867).

17. Naome MM, Rugare KL, Michelle DN, et al. Determinants of loss to follow-up in patients on antiretroviral treatment, South Africa, 2004-2012: a cohort study. *BMC Health Services Research* 2015;15(1):1.

18. Wilson TE, Hogben M, Malka ES et al. A randomized controlled trial for reducing risks for sexually transmitted infections through enhanced patient-based partner notification. *Am J Public Health* 2009;99(1):104-10.

19. FMOH. Syndromic managment of sexually transmited infections 2015.

20. Alam N, Streatfield PK, Khan SI, et al. Factors associated with partner referral among patients with sexually transmitted infections in Bangladesh. *Soc Sci Med.* 2010 71(11):1921-6.

21. Tigray Regional Health Bureau. Measuring index score of health facility in Tigray regional state. In: office r, editor, 2015.

22. Pickering JM, Whitworth J A G, Hughes P, et al. Aetiology of sexually transmitted infections and response to syndromic treatment in southwest Uganda. *Sex Transm Infect* 2005;81:488-93.

23. Stephenson R. Community-Level Gender Equity and Extramarital Sexual Risk-Taking Among Married Men in Eight African Countries. *International Perspectives on Sexual and Reproductive Health* 2010;36(4):178-88.

24. Rahman AA, Rahman RA, Ismail SB, et al. Factors associated with attitude toward premarital sexual activities among school-going adolescents in Kelantan, Malaysia. *Asia Pac J Public Health* 2015 27(2):1549-56.

25. Tucker JS, Burnam MA, Sherbourne CD, et al. Substance use and mental health correlates of non-adherence to antiretroviral medications n a sample of patients with human immune deficiency virus infection. . *Am J Med.* 2003;114(7):573-80.

26. Mmbaga EJ, Leyna GH, Mnyika KS et al. Sexually transmitted infections knowledge and its impact in the practice of risky sexual behaviours and HIV serostatus: results from rural kilimanjaro, Tanzania. *Sex Transm Infect.* 2008 84(3):224-6.

For peer review only

27. Morris JL, Lippman SA, Philip S, et al. Sexually Transmitted Infection Related Stigma and Shame Among African American Male Youth: Implications for Testing Practices, Partner Notification, and Treatment. *AIDS Patient Care STDS* 2014 28(9):499-506.

28. Bwirire LD, Fitzgerald M, Zachariah R, et al. Reasons for loss to follow-up among mothers registered in a prevention-of-mother-to-child transmission program in rural Malawi. *Trans R Soc Trop Med Hyg* 2008;102(12):1195-200.

29. Brinkhoft MW, Dabis F, Myer L, et al. Earlyloss of HIV-infected patients on potent antiretroviral therapy programmes in lowerincome countries. *Bulletin of World Health Organization* 2008;86:559-67.

30. Ojikutu B, Higgins-Biddle M, Greeson D, et al. The Association between Quality of HIV Care, Loss to Follow-Up and Mortality in Pediatric and Adolescent Patients Receiving Antiretroviral Therapy in Nigeria. *PLoS ONE* 2014;9(7).

For peer review only

Table-1: Description and measurements of variables in the models, North Ethiopia, 2015

Variable	*Description*	*Measurement*
Type of partnership	The relationship of index case with sexual partner (*Regular*: if there is ongoing relationship for ≥3 months. (*Casual*: if the relationship is <3 months).	Regular, Casual
STI transmission	Patient's knowledge of STI transmission: *Mean score(SD)= 2.74(1.69), 5 items*	≥Mean=Good; <Mean=poor
STI symptoms	Patient's knowledge of STI symptoms: *Mean score(SD) = 4.03(2.04), 6 items*	≥Mean=Good; <Mean=poor
STI prevention	Patient's knowledge of STI prevention: M*ean score(SD*) =4.62(1.00), 5 items	≥Mean=Good; <Mean=poor
STI Complication	Patient's knowledge of STI complication: *Mean score(SD) =2.01(1.84), 5 items*	≥Mean=Good; <Mean=poor
Perceived stigma	Patient's perceived stigma to PN: *Mean score(SD) = 12.92(2.52), 4 items*	≥Mean=high ; <Mean=low
HFI*	Health facility index score	High(>75), Medium (50-74.9), Low (<50)
HFS*	Health facility setting	Urban, Rural
Distance***	Walking distance of HF from home	≤1hrs walk, >1hrs walk
STI trained**	Availability of trained care provider in STIs	Yes, No
STI patient flow*	Annual STI pt flow to health facility: *Mean score(SD) =166.26(85)*	≥Mean=high ; <Mean=low
Guideline**	Availability of guidelines	Yes, No

*Health management information system (HMIS) = Data from Regional Health Bureau

**Facility assessment

***patients interview

HFI=According to HMIS report, none of the selected health facility had "low" category.

For peer review only

Table-2: Profile of study subjects, North Ethiopia, 2015

Characteristic	*Entire cohort (%)*	*Loss to follow-up*	
		Yes (%)	***No (%)***
Sex			
Female	599(55.36)	304(50.7)	295(49.3)
Male	483(44.64)	343(71.0)	140(29.0)
Age			
<25 yrs	544(50.28)	326(59.9)	218(40.1)
>=25 yrs	538(49.72)	321(59.6)	217(40.4)
Education			
Illiterate	190(17.56)	140(73.6)	50(26.4)
Primary	324(29.94)	197(60.8)	127(39.2)
Secondary	397(36.69)	219(55.1)	178(44.9)
College +	171(15.80)	91(53.2)	80(46.8)
Marital status			
Married	516(47.69)	264(51.2)	252(48.8)
Single	566(52.31)	383(67.7)	183(32.3)
Residence			
Urban	805(74.39)	486(60.4)	319(39.6)
Rural	277(25.61)	161(58.1)	116(41.9)
Type of partnership			
Regular	636(58.78)	346(54.4)	290(45.6)
Casual	446(41.22)	301(67.5)	145(32.5)
No partners last 3 months			
One	938(86.69)	533(56.8)	405(43.2)
Two or more	144(13.31)	114(79.2)	30(20.8)
Perceived stigma to PN			
High	390(36.04)	248(63.6)	142(39.4)
Low	692(63.96)	399(57.6)	293(42.4)
Distance from health facility			
<1hr walk	824(76.16)	450(54.6)	374(45.4)
≥1hr walk	258(23.84)	197(76.4)	61(23.6)
Health facility index score			
High	346(32.00)	176(50.8)	170(49.2)
Medium	736(68.00)	471(64.0)	265(36.0)
Health facility setting			
Urban	794(73.38)	479(60.3)	315(39.7)
Rural	288(26.62)	168(58.3)	120(41.7)
Patient flow to health facility			
Low	668(61.73)	344(51.5)	344(51.5)
High	414(38.27)	303(73.2)	303(73.2)

For peer review only - http://bmjopen.bmj.com/site/about/guidelines.xhtml

For peer review only

Table-3: Multivariable Multilevel logistic regression analysis of individual and HF level factors associated with LTFU in North Ethiopia, 2015.

Characteristics	*Model-1*	*Model-2* *AOR (95%CI)*	*Model-3* *AOR (95%CI)*	*Model-4* *AOR (95%CI)*
Individual level variables				
Sex of index case				
Female		1		1
Male		2.05(1.48-2.82)*		2.23(1.63-3.04)*
Marital status				
Married		1		
Single		1.36(0.95-1.95)		
Educational status				
Illiterate		1		1
Primary		1.07(0.68-1.68)		1.11(0.71-1.74)
Secondary		0.85(0.54-1.33)		0.89(0.57-1.38)
College +		0.38(0.22-0.64)*		0.38(0.22- 0.65)*
Type of partnership				
Regular		1		
Casual		0.74(0.72-1.58)		
Number of partner last 3months				
One		1		1
Two or more		2.81(1.69-4.67)*		2.89(1.74-4.80)*
Knowledge of STI transmission				
Good		1		1
poor		2.09(1.54-2.85)*		2.08(1.53-2.82)*
Knowledge of STI complication				
Good		1		1
poor		1.53(1.13-2.09)*		1.56(1.15-2.12)*
Perceived stigma				
High		1		1
Low		0.61(0.44-0.83)*		0.60(0.43-0.82)*
Health Facility level variables				
Health facility index				
High			1	1

For peer review only - http://bmjopen.bmj.com/site/about/guidelines.xhtml

Medium			2.85(1.38-5.86)*	2.80(1.28-6.13)*
STI patient flow				
Low			1	1
High			3.00(1.37-6.57)*	3.06(1.30-7.18)*
Random effect parameters				
Variance	1.04*	1.15*	0.62*	0.74*
ICC (%)	24.12	25.90	15.85	18.56
PCV (%)	Ref	10.57	40.38	27.88
Model fitness				
Log likelihood	-660.74	-583.66	-625.08	-577.92
AIC	1325.48	1167.32	1250.16	1155.84

**P-value<0.05; AOR=Adjusted odds ratio; ICC=Intra class correlation coefficient. 1=reference*

PCV=Proportional change in variance, AIC=Akaike information criterion

Model-1=null model

Model-2=adjusted for individual-level factors alone

Model-3=adjusted for facility-level factors alone

Model-4=adjusted for both individual and facility-level factors

For peer review only - http://bmjopen.bmj.com/site/about/guidelines.xhtml

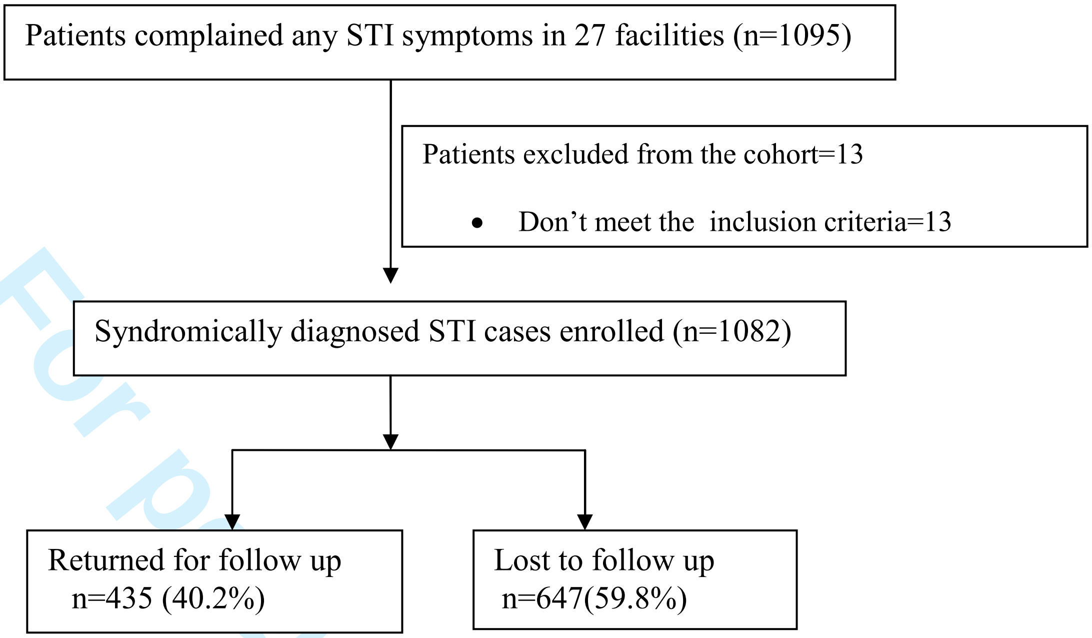

Figure 1: The proportion of LTFU cases among patients attended for STI care, North Ethiopia, 2015.

For peer review only - http://bmjopen.bmj.com/site/about/guidelines.xhtml

STROBE 2007 (v4) Statement—Checklist of items that should be included in reports of *cohort studies*

Section/Topic	Item #	Recommendation	Reported on page #
**Title and abstract**	1	(*a*) Indicate the study's design with a commonly used term in the title or the abstract	1,2
		(*b*) Provide in the abstract an informative and balanced summary of what was done and what was found	2
**Introduction**			
Background/rationale	2	Explain the scientific background and rationale for the investigation being reported	4
Objectives	3	State specific objectives, including any prespecified hypotheses	4
**Methods**			
Study design	4	Present key elements of study design early in the paper	5
Setting	5	Describe the setting, locations, and relevant dates, including periods of recruitment, exposure, follow-up, and data collection	5
Participants	6	(*a*) Give the eligibility criteria, and the sources and methods of selection of participants. Describe methods of follow-up	5
		(*b*) For matched studies, give matching criteria and number of exposed and unexposed	=
Variables	7	Clearly define all outcomes, exposures, predictors, potential confounders, and effect modifiers. Give diagnostic criteria, if applicable	5, 6,13
Data sources/ measurement	8*	For each variable of interest, give sources of data and details of methods of assessment (measurement). Describe comparability of assessment methods if there is more than one group	*13*
Bias	9	Describe any efforts to address potential sources of bias	5
Study size	10	Explain how the study size was arrived at	5
Quantitative variables	11	Explain how quantitative variables were handled in the analyses. If applicable, describe which groupings were chosen and why	6
Statistical methods	12	(*a*) Describe all statistical methods, including those used to control for confounding	6
		(*b*) Describe any methods used to examine subgroups and interactions	6
		(*c*) Explain how missing data were addressed	-
		(*d*) If applicable, explain how loss to follow-up was addressed	5
		(*e*) Describe any sensitivity analyses	-
**Results**			

For peer review only

Participants	13*	(a) Report numbers of individuals at each stage of study—eg numbers potentially eligible, examined for eligibility, confirmed eligible, included in the study, completing follow-up, and analysed	7
		(b) Give reasons for non-participation at each stage	Not applicable
		(c) Consider use of a flow diagram	18
Descriptive data	14*	(a) Give characteristics of study participants (eg demographic, clinical, social) and information on exposures and potential confounders	7
		(b) Indicate number of participants with missing data for each variable of interest	
		(c) Summarise follow-up time (eg, average and total amount)	7
Outcome data	15*	Report numbers of outcome events or summary measures over time	7
Main results	16	(*a*) Give unadjusted estimates and, if applicable, confounder-adjusted estimates and their precision (eg, 95% confidence interval). Make clear which confounders were adjusted for and why they were included	7
		(*b*) Report category boundaries when continuous variables were categorized	
		(*c*) If relevant, consider translating estimates of relative risk into absolute risk for a meaningful time period	
Other analyses	17	Report other analyses done—eg analyses of subgroups and interactions, and sensitivity analyses	
**Discussion**			
Key results	18	Summarise key results with reference to study objectives	8
**Limitations**			
Interpretation	20	Give a cautious overall interpretation of results considering objectives, limitations, multiplicity of analyses, results from similar studies, and other relevant evidence	8, 9
Generalizability	21	Discuss the generalizability (external validity) of the study results	9
**Other information**			
Funding	22	Give the source of funding and the role of the funders for the present study and, if applicable, for the original study on which the present article is based	10

*Give information separately for cases and controls in case-control studies and, if applicable, for exposed and unexposed groups in cohort and cross-sectional studies.

Note: An Explanation and Elaboration article discusses each checklist item and gives methodological background and published examples of transparent reporting. The STROBE checklist is best used in conjunction with this article (freely available on the Web sites of PLoS Medicine at http://www.plosmedicine.org/, Annals of Internal Medicine at http://www.annals.org/, and Epidemiology at http://www.epidem.com/). Information on the STROBE Initiative is available at www.strobe-statement.org.

For peer review only

The magnitude and factors associated with loss to follow-up among patients treated for sexually transmitted infections: A multilevel analysis

Journal:	*BMJ Open*
Manuscript ID	bmjopen-2017-016864.R1
Article Type:	Research
Date Submitted by the Author:	22-May-2017
Complete List of Authors:	Tsadik, Mache; Mekelle University<br>Berhane, Yemane ; Addis Continental Institute of Public Health<br>Worku, Alemayehu; Addis Ababa University<br>Terefe, Wondwossen; Mekelle University
**Primary Subject Heading**:	Infectious diseases
Secondary Subject Heading:	Public health, Sexual health, Epidemiology
Keywords:	PUBLIC HEALTH, Infection control < INFECTIOUS DISEASES, Epidemiology < INFECTIOUS DISEASES

SCHOLARONE™
Manuscripts

For peer review only

The magnitude and factors associated with loss to follow-up among patients treated for sexually transmitted infections: A multilevel analysis

Mache Tsadik [1*,] Yemane Berhane[2], Alemayehu Worku[3], Wondwossen Terefe[1]

Mache Tsadik (adhana2008@gmail.com)

Yemane Berhane (yemaneberhane@gmail.com)

Alemayehu Worku (alemayehuwy@yahoo.com)

Wondwossen Terefe (wlerebo@uwc.ac.za)

Affiliation

1 College of health science, Mekelle University, Tigray, Ethiopia

2 Addis Continental Institute of Public Health, Addis Ababa, Ethiopia

3 Addis Ababa University, Addis Ababa, Ethiopia

* Corresponding Author, adhana2008@gmail.com

For peer review only

For peer review only - http://bmjopen.bmj.com/site/about/guidelines.xhtml

Abstract

Objectives: The lost to follow up among patients attending STI care in Sub-Saharan Africa is major barrier to achieving the goals of the STI prevention and control program. The objective of this study was to investigate individual- and facility-level factors associated with lost to follow-up (LTFU) among patients treated for sexually transmitted infections (STIs) in Ethiopia.

Methods: A prospective cohort study was conducted among patients attending care for STIs in selected facilities from January to June 2015 in Tigray region of Ethiopia. LTFU was ascertained if a patient didn't present in person to the same facility within seven days of the initial contact. Multilevel logistic regression was used to identify factors associated with LTFU.

Results: Out of 1082 patients, 59.80% (647) were LTFU. The individual-level factors associated with LTFU included having multiple partners (AOR=2.89, 95%CI: 1.74-4.80), being male (AOR=2.23, 95%CI: 1.63-3.04), having poor knowledge about the means of STI transmission (AOR=2.08, 95%CI: 1.53-2.82), having college level education (AOR=0.38, 95%CI: 0.22-0.65) and low perceived stigma (AOR=0.60, 95% CI: 0.43-0.82). High patient flow (AOR=3.06, 95%CI: 1.30-7.18) and medium health index score (AOR=2.80, 95%CI: 1.28-6.13) were facility-level factors associated with LTFU.

Conclusions: Improving patient retention in STI follow up care requires focused interventions targeting those who are more likely to be LTFU, particularly patients with multiple partners, male index cases and patients attending facilities with high patient flow.

Key words: sexually transmitted infections, partner notification, loss to follow-up, multilevel analysis

For peer review only

Strengths and limitations of this study

- This study generated useful information that can help improve the clinical and public health interventions in the management of STIs and in reducing LTFU.
- The study used a prospective cohort design that has the potential to minimize biases related to other observational study designs.
- Advanced statistical analytic model that allow simultaneous examination of individual and facility level factors that can affect the follow up care of patients attending STI care.
- The specific reasons for LTFU were not identified because there was no established participant tracing mechanism for patients who were LTFU in the context of this study.
- The study included only STI patients attending public health facilities thus inference to patients seeking care in private facilities requires careful considerations of the local context.

For peer review only

For peer review only - http://bmjopen.bmj.com/site/about/guidelines.xhtml

Introduction:

The prevalence and incidence of sexually transmitted infections (STIs) in Sub-Saharan Africa are among the highest in the world.[1] Due to shortcomings related to laboratory capacity STI prevention and control programs adapted a syndromic management in which partner notification is a key component of treatment package.[2-4] Because of the risk of reinfection treating partner(s) of the index case as early as possible is critical.[5] Thus it is important to follow index cases to assure compliance to treatment, and ascertain partner notification status [6 7] in order to effectively reduce the burden of STIs. [5 8] However, lost to follow-up (LTFU) has remained one of the challenges to effectively implement the existing treatment and preventive strategies and information on LTFU from Sub-Saharan Africa is scanty.

A number of factors have been linked to LTFU in the management of STIs cases. LTFU is more likely among males [9], single (not in union) index cases [10], individuals with low level of education compared to higher level of education [11], individuals with poor knowledge of STIs [12], and individuals who did not intend to notify partners.[13] Moreover, reluctance to return to the same facility for follow up due to fear of negative judgments.[14] On the provider side, poor quality of health services including inadequate patient education and lack of follow-up advice [15], judgmental approach of care providers, and lack of privacy and confidentiality contributes to LTFU. [10 16-18]

This study is conducted in public health facilities of Tigray region, north Ethiopia where little information is known about the magnitude of LTFU among patients treated for STIs and the associated factors. The public health facilities implement the national syndromic management protocol and treatment is provided in low charge. Therefore, the aim of this study was to investigate individual and facility-level factors associated with LTFU among STI patients attending public health facilities in North Ethiopia.

For peer review only - http://bmjopen.bmj.com/site/about/guidelines.xhtml

Methods:

The study was conducted in public health facilities of Tigray regional state, north Ethiopia. We selected health facilities with monthly patient load (STIs) of five and above in order to make the research project manageable with the resources available for the study. Thus, of the 108 public health facilities in the study area 27 fulfilled the study selection criteria. According to the national guideline, STI syndromes include vaginal discharge, urethral discharge, genital ulcer, lower abdominal pain, scrotal swelling, inguinal bubo and neonatal conjunctivitis.[19] Thus patients presenting with complaints such as burning sensation, genital discharge, genital ulcer and other related symptoms were treated as cases of STI using the Syndromic Management protocol that was adapted from the WHO generic protocol.

We conducted a prospective cohort study among patients attending public health facilities for STI care. Self-referred patients with one or more of the STI syndromes and who had sexual intercourse within three months preceding the study were recruited as study subjects. All patients that come seeking treatment for STI related symptoms during the study period were included in the study. The research team in collaboration with care providers ensured that patients receive follow-up advice and appointment. A baseline interview was then conducted to collect relevant information from each patient by trained research nurses in a private room after obtaining an informed consent. Patients were informed to notify their partners and return for follow-up within 7-days. Patients received instructions and information card containing facility room where they should return and contact details of the research assistant to facilitate their follow-up visit. A minimum of two research assistants was also assigned to a health facility to capture patients returned for follow-up within the specified period.

Sample size for the study was calculated using two proportion sample size formula to determine predictors of PN for STIs by considering 40% of married individuals who notified partners [20] and the following assumptions were used: odds ratio of 1.5, 80% power with 95% CI and 20% non response rate.

The study tool was developed by reviewing relevant literature and then adapting to the context of our study. At the individual level, the tool comprises of sociodemographic, behavioral and psychosocial components. Some of the factors considered at facility level were distance, trained providers, availability of treatment guideline, patient flow, and health index score. The tool was

For peer review only - http://bmjopen.bmj.com/site/about/guidelines.xhtml

pre-tested before the actual use in facilities not included in the study. The study measurements were defined and described in table 1. According to the Health Management Information System (HMIS) report of Tigray Regional Health Bureau, public health facilities were classified into three levels using the health facility index score as low (<50), medium (50-74.9) and high (≥75).[21] However, among the facilities selected for this study no health facility belonged in the "low" category.

Statistical analysis:

LTFU was ascertained if the index case fails to return to the same health facility within 7-days of the initial clinic visit; and patients who were not LTFU were referred to as "in follow up care". LTFU was categorized as "Yes" for those LTFU and "No" for those retained in care.

Both individual and facility-level variables were described and presented using a simple frequency table. Before multivariate analysis was performed, Pearson's chi-square tests were used to check for the crude association between the dependent variable (LTFU) and individual and facility level variables. Then, all independent variables with *P*-value smaller than the significance level (0.05) were entered into the model.

In multilevel analyses, a null model with no covariates was used to assess the presence of significant clustering in LTFU. For individual-level factors the analysis considered sex, marital status, education, the number of partners, type of partnership, knowledge of STI transmission methods and complication, and perceived stigma. Facility-level variables include health facility index and STI patient flow. The command "xtmelogit" was used to fit a mixed-effect multilevel logistic regression model and the relationship between the dependent variable and each of the independent variables (i.e. fixed effects) were assessed using odds ratios and their confidence intervals. To evaluate the significance of facility-level clustering of the dependent variable (i.e. random effects), log-likelihood ratio tests were employed. Collinearity between variables was assessed by looking at the values of variance inflation factors (VIF), and the mean correlation value in the fitted model was 2.02.

For peer review only

Results:

A total of 1082 patients who received STI care in selected health facilities were enrolled in the study. Of which, 647 (59.80%, 95% CI: 56.88-62.72) patients who did not return for follow-up care within seven days were categorized as LTFU.

Patient and facility-level characteristics

Patient and facility level characteristics of the study sample are presented in table 2. The mean age of the cohort population was 26.4 years (SD=7.6). More than 50% of patients presented with STIs had at least high school level education. A substantial number of patients (41.22%) reported casual partnership. The majority of the health facilities (73.38%) were located in the urban settings. About three-fourth of patients reported residing within one-hour walking distance from the health facility they visited. The large proportion of LTFU (73.2%) was observed in health facilities with the high patient flow.

***Multilevel logistic regression analysis*:**

After controlling the potential confounders at individual and facility-level, the odds of LTFU were greater among index cases with multiple partners (AOR=2.89, 95%CI: 1.74-4.80), males (AOR=2.23, 95%CI: 1.63, 3.04), individuals with poor knowledge of STI transmission (AOR=2.08, 95%CI: 1.53-2.82) and poor knowledge of STI complication (AOR=1.56, 95% CI: 1.15-2.12). LTFU was less likely among better educated individuals (AOR=0.38, 95%CI: 0.22-0.65) and those with perceived low stigma (AOR=0.60, 95%CI: 0.43-0.82). LTFU was more likely among patients who received care in facilities with high patient flow were three times (AOR=3.06, 95%CI: 1.30-7.18) and among patients that received care in facilities with medium health index score compared to highest index score (AOR=2.80, 95%CI: 1.28-6.13) (Table 3).

For peer review only - http://bmjopen.bmj.com/site/about/guidelines.xhtml

Discussion:

About two-third (59.8%) of STI patients were lost to follow-up in this study. The individual level factors associated with increased likelihood of LTFU were being male, having multiple sexual partnerships and having poor knowledge about the means of STI transmission and their complications. While those who achieved a higher level of education and reported low perceived stigma were less likely to be LTFU. The odds of LTFU were greater among patients seen in health facility with medium health index score and in facilities with high patient flow.

The level of LTFU among STI patients is similar to other studies.[10 18] Early response to treatment, relief of symptoms and fear of stigma on return to follow-up were identified as factors contributing to LTFU.[22 23] Our study clearly indicates the potential for reinfection is quite high and that may in turn facilitate the development of drug resistance STI. In addition, since a substantial proportion of cases reported multiple sexual partnerships those untraceable and probably re-infected would continue spreading STIs in the community. This high proportion of LTFU is a major challenge to the STI prevention and control efforts and need to be addressed urgently.

The study identified a number of independent LTFU predictors both in individual and facility-level factors. In this study, males were more likely to be LTFU compared to females. This finding was consistent with studies conducted in Uganda,[24] Malawi [25] and South Africa.[26] As suggested by Geng et al., males are more likely to use substances that potentially decreases their adherence to follow-up care.[27] Males also report low stigma in relation to their engagement in risky sexual behaviors.[28]

The less likelihood of LTFU among educated individuals in this study is consistent with a previous study.[10]. [29],[30] This may suggest that education is an important factor in adhering to medical care.[31] Besides, highly educated individuals have a higher knowledge of STIs and individuals with high knowledge of STIs are less likely to be LTFU [12] may be because of fear of the subsequent complications. The motivation to attend follow-up care among educated individuals may be associated with their understanding of potential benefits.

LTFU was less likely among patients with low perceived stigma compared to those reported perceived high stigmas. This may show that individuals with low perceived stigma are confident

For peer review only - http://bmjopen.bmj.com/site/about/guidelines.xhtml

enough to notify partners and have the courage to return for follow-up.[32] The stigma linked to STIs reduces the motive and willingness of index cases to notify partners and resulted in greater LTFU.[33] Fear of provider's judgmental reactions during follow-up care and embracement negatively affects follow-up considerably in low income settings.[34]

LTFU is higher among patients from facilities with a high patient flow in the present study. A similar observation was reported previously in HIV treatment setting where high patient load was associated with high proportion of LTFU.[35] The high patient load might limit the provider's time to provide adequate care that potentially influences index cases to LTFU.[36] The odds of LTFU were also lower among patients attending facilities with high index score in our study. Similarly, patient focused study conducted in Nigeria has shown that patients with high quality score were less likely to LTFU.[37] This may indicate that high quality care motivates patients to remain in follow-up care.

This study has some important limitations. First, reasons for LTFU were not documented as this study did not have the resources to establish participant tracing mechanisms. Thus, patients who decide to do their follow up care at another health facility because of STI linked stigma may have been considered as LTFU. Second, study participants who returned for follow-up after the scheduled time were not captured. Third, since the study was conducted among self-referred patients from public health facilities, extrapolation of results to all STI patients should be made with caution since the factors related to LTFU among those seeking care in private facilities may not be similar. Behavior related information was all self-reported and may have some reliability issues because of social desirability and recall bias.

Despite the stated limitations, this study is one of the few studies ever conducted on LTFU among patients treated for STIs in our setting and we believe the information reported is helpful to improve interventions being implemented to prevent and control STIs in contexts similar to our study. This study also employed advanced method of analysis that allows the simultaneous analysis of individual and facility-level factors.

Conclusion:

Overall, the magnitude of LTFU among patients being treated for STIs is very high in North Ethiopia. The need for standardized follow up care and cost effective tracing mechanism is important to retain and trace STI patients who are at risk of LTFU.

For peer review only - http://bmjopen.bmj.com/site/about/guidelines.xhtml

For peer review only

Acknowledgement

We would like to acknowledge Mekelle University and Addis Continental Institute of Public Health for the overall technical and material support. This research was partially funded by African Doctoral Dissertation Research Fellowship award (ADDRF) (Grant Number: ADF 015). We would also like to thank the Tigray Health Bureau and directors of the study health facilities for facilitating the conduct of this study. We are also grateful to all study participants, data collectors and supervisors without whom this work would not have been possible.

Authors' contribution

The study was designed by MT, YB, AW and WT. MT was responsible for data collection, analysis and drafting the manuscript. YB reviewed the study design and revised the manuscript. AW supervised the data collection and analysis, and revising the manuscript, and contributed to interpretation of the analysis. WT participated in the analysis and interpretation of the data, and revised the manuscript. All authors have read and approved the final manuscript.

Funding: The study is funded by Mekelle University and African Population Health Research Center (APHRC). The funders had no role in the gathering or analysis of the data and no role in the writing of the manuscript or the decision to submit for publication.

Competing interests: The authors declare that they have no competing interests.

Ethical Approval*:* Mekelle University Health Research Ethical Review Committee (HRERC) gave ethical approval. ERC 0458/2014, date approved: 01 November 2014. The study participants gave informed consent before being involved in the study.

Data sharing statement*:* No additional data available

For review only

Reference:

1. World Health Organization (WHO). Global Prevalence and Incidence of Selected Curable Sexually Transmitted Infections: , 2012.
2. Golden MR, Faxelid E, and Low N. Partner notification for sexually transmitted infections including HIV infection: an evidence-based assessment.New York: McGraw-Hill. *Sex transm dis.* 2008:965-84.
3. Low N, Hawkes SJ. Putting the magic in magic bullets: top three global priorities for sexually transmitted infection control. *Sex Transm Infect.* 2011;87:44-46.
4. Centers for Disease Control and Prevention. Recommendations for Partner Services Programs for HIV Infection, Syphilis, Gonorrhea, and Chlamydial Infection. *MMWR* 2008;57. (RR-9):1-92.
5. Low N, Heijne JC, Herzog SA, et al. Reinfection by untreated partners of people treated for Chlamydia trachomatis and Neisseria gonorrhoeae: mathematical modelling study. *Sex Transm Infect* 2014;90(3):254-6.
6. Azariah S. Partner notification for sexually transmitted infections. Why can't we talk about it? *Journal of the New Zealand Medical Association* 2012;125(1355).
7. Lazaro N. Sexually Transmitted Infections in Primary Care 2013 (RCGP/BASHH). available at www.rcgp.org and www.bashh.org/guidelines, 2013.
8. Bell G, Potterat J. Partner notification for sexually transmitted infections in the modern world: a practitioner perspective on challenges and opportunities. *Sex Transm Infect. 2011; 87:34-36 doi:10.1136/sextrans-2011-050229* 2011.
9. Burnet Institute. Partner notification of sexually transmitted infections in New South Wales: An informed literature review., 2010.
10. Tang W, Huan X, Zhang Y et al. . Factors Associated with Loss-toFollow-Up during Behavioral Interventions and HIV Testing Cohort among Men Who Have Sex with Men in Nanjing, China. *PLoS ONE* 2015;10(1):e115691.
11. Ahmed I, Gugsa ST, Lemma S, et al. Predictors of loss to follow-up before HIV treatment initiation in Northwest Ethiopia: a case control study. *BMC Public Health* 2013;13(867).
12. Naome MM, Rugare KL, Michelle DN, et al. Determinants of loss to follow-up in patients on antiretroviral treatment, South Africa, 2004-2012: a cohort study. *BMC Health Services Research* 2015;15(1):1.
13. Wilson TE, Hogben M, Malka ES et al. A randomized controlled trial for reducing risks for sexually transmitted infections through enhanced patient-based partner notification. *Am J Public Health* 2009;99(1):104-10.
14. Ware NC WM, Geng EH, Kaaya SF, Agbaji OO, Muyindike WR, et al. Toward an understanding of disengagement from HIV treatment and care in Sub-saharan Africa: A qualitative study. *PLOS medicine* 2013;10(1):e1001369.
15. Wu H, Yim, C., Chan, A., Ho, M., and Heathcote, J. Sociocultural factors that potentially affect the institution of prevention and treatment strategies for prevention of hepatitis B in Chinese Canadians. *Can J Gastroenterol* 2009;23: 31-36.
16. Malik AI, Huppert JS. Interval to treatment of sexually transmitted infections in adolescent females. *J Pediatr Adolesc Gynecol* 2007;20(5):275-79.
17. Huppert J, Reed JL, Munafo JK, et al. Improving Notification of Sexually Transmitted Infections: A Quality Improvement Project and Planned Experiment. *PEDIATRICS* 2012;130(2):415-22.

For peer review only - http://bmjopen.bmj.com/site/about/guidelines.xhtml

18. Buchsbaum A, Gallo MF, Whiteman MK,. Sexually Transmitted Disease Partner Notification among African-American, Adolescent Women. *Infectious Diseases in Obstetrics and Gynecology* 2014;(2014).
19. FMOH. Syndromic managment of sexually transmited infections 2015.
20. Alam N, Streatfield PK, Khan SI, et al. Factors associated with partner referral among patients with sexually transmitted infections in Bangladesh. *Soc Sci Med* 2010;71(11):1921-6.
21. Tigray Regional Health Bureau (TRHB). Measuring index score of health facility in Tigray regional state, 2015.
22. Pickering JM, Whitworth J A G, Hughes P, et al. Aetiology of sexually transmitted infections and response to syndromic treatment in southwest Uganda. *Sex Transm Infect* 2005;81:488-93.
23. CDC. Sexually Transmitted Diseases Treatment Guidelines, 2015.
24. Amuron B, Namara G, Birungi J et al. Nabiryo C, Levin J, Grosskurth H, Coutinho A, Jaffar S. Mortality and loss-to-follow-up during the pre-treatment period in an antiretroviral therapy programme under normal health service conditions in Uganda. *BioMed Central* 2009;9:290.
25. Zachariaha R T-SK, Manzi M et al. Massaquoi M, Mwagomba B, Griensven J, Engelgem I, Arnould L, Schouten EJ, Chimbwandira FM, Harries AD. Retention and attrition during the preparation phase and after start of antiretroviral treatment in Thyolo, Malawi, and Kibera, Kenya: implications for programmes? . *Trans R Soc Trop Med Hyg* 2011;4191:10.
26. Cornell M, Grimsrud A, Fairall L, Fox MP, van Cutsem G, et al. Temporal changes in programme outcomes among adult patients initiating antiretroviral therapy across South Africa, 2002–2007. *Aids* 2010;24: 2263-2270.
27. Geng EH, Bangsberg DR, Musinguzi N, et al. Understanding reasons for and outcomes of patients lost to follow-up in antiretroviral therapy programs in Africa through a sampling-based approach. *J Acquir Immune Defic Syndr* 2010;53(3):405-11.
28. Stephenson R. Community-Level Gender Equity and Extramarital Sexual Risk-Taking Among Married Men in Eight African Countries. *International Perspectives on Sexual and Reproductive Health* 2010;36(4):178-88.
29. Panditrao M, Darak S, Kulkarni V, Kulkarni S, Parchure R. Socio-demographic factors associated with loss to follow-up of HIV-infected women attending a private sector PMTCT program in Maharashtra, India. *AIDS Care* 2011;23(5):593-600.
30. Ruan Y, Jia Y, Zhang X, Liang H, Li Q, et al. Incidence of HIV-1, syphilis, hepatitis B, and hepatitis C virus infections and predictors associated with retention in a 12-month follow-up study among men who have sex with men in Beijing, China. . *Journal of Acquired Immune Deficiency Syndromes* 2009;52: 604-610.
31. Tucker JS, Burnam MA, Sherbourne CD, et al. Substance use and mental health correlates of non-adherence to antiretroviral medications n a sample of patients with human immune deficiency virus infection. *Am J Med* 2003;114(7):573-80.
32. Megerso A, Garoma S, Eticha T, et al. Predictors of loss to follow-up in antiretroviral treatment for adult patients in the Oromia region, Ethiopia. *HIV/AI DS - Research and Palliative Care* 2016:8.
33. Morris JL, Lippman SA, Philip S, et al. Sexually Transmitted Infection Related Stigma and Shame Among African American Male Youth: Implications for Testing Practices, Partner Notification, and Treatment. *AIDS Patient Care STDS* 2014;28(9):499-506.

For peer review only - http://bmjopen.bmj.com/site/about/guidelines.xhtml

34. Bwirire LD, Fitzgerald M, Zachariah R, et al. Reasons for loss to follow-up among mothers registered in a prevention-of-mother-to-child transmission program in rural Malawi. *Trans R Soc Trop Med Hyg* 2008;102(12):1195-200.
35. Brinkhof MW, Dabis F, Myer L, et al. Early loss of HIV-infected patients on potent antiretroviral therapy programmes in low-income countries. *Bulletin of World Health Organization* 2008;86:559-67.
36. Van Damme W, Kober K, Kegels G. Scaling-up antiretroviral treatment in Southern African countries with human resource shortage: How will health systems adapt? . *Social science & medicine* 2008;66(10):2108-21.
37. Ojikutu B, Higgins-Biddle M, Greeson D, et al. The Association between Quality of HIV Care, Loss to Follow-Up and Mortality in Pediatric and Adolescent Patients Receiving Antiretroviral Therapy in Nigeria. *PLoS ONE* 2014;9(7).

For peer review only

For peer review only - http://bmjopen.bmj.com/site/about/guidelines.xhtml

Table-1: Description and measurements of variables in the models, North Ethiopia, 2015

Variable	***Description***	***Measurement***
Type of partnership	The relationship of index case with sexual partner (*Regular*: if there is ongoing relationship for ≥3 months. (*Casual*: if the relationship is <3 months).	Regular,<br>Casual
STI transmission	Patient's knowledge of STI transmission: *Mean score(SD)= 2.74(1.69), 5 items*<br>1. Unprotected sexual intercourse 4. Blood transfusion<br>2. Mother to child during birth 5.Breast feeding<br>3. Injury by sharp materials (needle, blade)<br>* *Response category (yes, no, I don't know) (Cronbach's Alpha =0.77)*	≥Mean=Good;<br><Mean=poor
STI symptoms	Patient's knowledge of STI symptoms: *Mean score(SD) = 4.03(2.04), 6 items*<br>1. Vaginal discharge 4.Pain on passing urine<br>2. Itchiness in genitalia 5.Ulcers in the gentalia<br>3. Pain/swelling in the groin 6.Eye discharge in newborn<br>* *Response category (yes, no, I don't know) (Cronbach's Alpha=0.84)*	≥Mean=Good;<br><Mean=poor
STI prevention	Patient's knowledge of STI prevention: M*ean score(SD*) =4.62(1.00), 5 items<br>1. Abstinence 4. Use of condom<br>2. Having single partner 5. Early treatment<br>3. Avoid sex with risk partners<br>* *Response category (yes, no, I don't know) (Cronbach's Alpha =0.81)*	≥Mean=Good;<br><Mean=poor
STI Complication	Patient's knowledge of STI complication: *Mean score(SD) =2.01(1.84), 5 items*<br>1. Caner of cervix 4. Infertility<br>2. Still birth 5. Ectopic pregnancy<br>3. Abortion<br>* *Response category (yes, no, I don't know) (Cronbach's Alpha =0.82)*	≥Mean=Good;<br><Mean=poor
Perceived stigma	Patient's perceived stigma to PN: *Mean score(SD) = 12.92(2.52), 4 items*<br>1. Referring a partner for STI diagnosis and treatment is shame<br>2. Attending health facility for STI treatment is embarrassed<br>3. A good man/women go to health facility for STI treatment<br>4. A good man/women notify his/her partner<br>* *Response category (Very likely, likely, unlikely, very unlikely)*<br>*(Cronbach's Alpha =0.73)*	≥Mean=high ;<br><Mean=low
HFI*	Health facility index score	High(>75),<br>Medium (50-74.9),<br>Low (<50)

For peer review only - http://bmjopen.bmj.com/site/about/guidelines.xhtml

HFS*	Health facility setting	Urban, Rural
Distance***	Walking distance of HF from home	≤1hrs walk,<br>>1hrs walk
STI trained**	Availability of trained care provider in STIs	Yes, No
STI patient flow*	Annual STI pt flow to health facility: *Mean score(SD) =166.26(85)*	≥Mean=high ;<br><Mean=low
Guideline**	Availability of guidelines	Yes, No

*Health management information system (HMIS) = Data from Regional Health Bureau

**Facility assessment

***patients interview

HFI=According to HMIS report, none of the selected health facility had "low" category.

For peer review only - http://bmjopen.bmj.com/site/about/guidelines.xhtml

Table-2: Profile of study subjects, North Ethiopia, 2015

Characteristic	*Loss to follow-up*		*Pearsons Chi-square (p-value)*
	Yes (%)	*No (%)*	
Gender			0.001
Female	304 (50.7)	295 (49.3)	
Male	343 (71.0)	140 (29.0)	
Age			0.930
<25 yrs	326 (59.9)	218 (40.1)	
>=25 yrs	321 (59.6)	217 (40.4)	
Education			0.001
Illiterate	140 (73.6)	50 (26.4)	
Primary	197 (60.8)	127 (39.2)	
Secondary	219 (55.1)	178 (44.9)	
College +	91 (53.2)	80 (46.8)	
Marital status			0.152
Married	264 (51.2)	252(48.8)	
Single	383 (67.7)	183(32.3)	
Residence			0.510
Urban	486 (60.4)	319 (39.6)	
Rural	161 (58.1)	116 (41.9)	
Type of partnership			0.001
Regular	346 (54.4)	290 (45.6)	
Casual	301 (67.5)	145 (32.5)	
No partners last 3 months			0.001
One	533 (56.8)	405 (43.2)	
Two or more	114 (79.2)	30 (20.8)	
Perceived stigma to PN			0.003
High	248 (63.6)	142 (39.4)	
Low	399 (57.6)	293 (42.4)	
Types of STI syndromes			0.069
Vaginal discharge	233 (44.21)	294 (55.79)	
Urethral discharge	128 (34.69)	241(65.31)	
Genital ulcer	32 (40.51)	47(59.49)	
Lower abdominal pain	28 (41.79)	39 (58.21)	

For peer review only - http://bmjopen.bmj.com/site/about/guidelines.xhtml

Others *	14 (35.00)	26 (65)	
Distance from health facility			0.443
<1hr walk	450 (54.6)	374 (45.4)	
≥1hr walk	197(76.4)	61 (23.6)	
Health facility index score			0.001
High	176 (50.8)	170 (49.2)	
Medium	471(64.0)	265 (36.0)	
Health facility setting			0.554
Urban	479 (60.3)	315 (39.7)	
Rural	168 (58.3)	120 (41.7)	
Patient flow to health facility			0.001
Low	324 (48.5)	344 (51.5)	
High	111(26.8)	303 (73.2)	

P value < 0.05 was considered statistically significant

For peer review only

Table-3: Multivariable Multilevel logistic regression analysis of individual and health facility level factors associated with LTFU North Ethiopia, 2015.

Characteristics	***Category***	***AOR (95%CI)***	***P-value***
Individual level variables			
Sex of index case			0.001
	Female	1	
	Male	2.23(1.63-3.04)	
Educational status			0.001
	Illiterate	1	
	Primary	1.11(0.71-1.74)	0.633
	Secondary	0.89(0.57-1.38)	0.613
	College +	0.38(0.22- 0.65)	0.001
Number of partner last 3months			0.001
	One	1	
	Two or more	2.89(1.74-4.80)	
Knowledge of STI transmission			0.001
	Good	1	
	Poor	2.08(1.53-2.82)	
Knowledge of STI complication			0.004
	Good	1	
	Poor	1.56(1.15-2.12)	
Perceived stigma			0.002
	High	1	
	Low	0.60(0.43-0.82)	
Health Facility level variables			
Health facility index			0.010
	High	1	
	Medium	2.80(1.28-6.13)	
STI patient flow			0.010
	Low	1	
	High	3.06(1.30-7.18)	
Variance		0.74*	

For peer review only

ICC (%)	18.56
PCV (%)	27.88
Model fitness	
Log likelihood	-577.92
AIC	1155.84

AOR=Adjusted odds ratio; ICC=Intra class correlation coefficient; 1=reference

*PCV=Proportional change in variance, AIC=Akaike information criterion * p value<0.05*

For peer review only

For peer review only - http://bmjopen.bmj.com/site/about/guidelines.xhtml

STROBE 2007 (v4) Statement—Checklist of items that should be included in reports of *cohort studies*

Section/Topic	Item #	Recommendation	Reported on page #
**Title and abstract**	1	(*a*) Indicate the study's design with a commonly used term in the title or the abstract	1,2
		(*b*) Provide in the abstract an informative and balanced summary of what was done and what was found	2
**Introduction**			
Background/rationale	2	Explain the scientific background and rationale for the investigation being reported	4
Objectives	3	State specific objectives, including any prespecified hypotheses	4
**Methods**			
Study design	4	Present key elements of study design early in the paper	5
Setting	5	Describe the setting, locations, and relevant dates, including periods of recruitment, exposure, follow-up, and data collection	5
Participants	6	(*a*) Give the eligibility criteria, and the sources and methods of selection of participants. Describe methods of follow-up	5
		(*b*) For matched studies, give matching criteria and number of exposed and unexposed	=
Variables	7	Clearly define all outcomes, exposures, predictors, potential confounders, and effect modifiers. Give diagnostic criteria, if applicable	5, 6,13
Data sources/ measurement	8*	For each variable of interest, give sources of data and details of methods of assessment (measurement). Describe comparability of assessment methods if there is more than one group	*13*
Bias	9	Describe any efforts to address potential sources of bias	5
Study size	10	Explain how the study size was arrived at	5
Quantitative variables	11	Explain how quantitative variables were handled in the analyses. If applicable, describe which groupings were chosen and why	6
Statistical methods	12	(*a*) Describe all statistical methods, including those used to control for confounding	6
		(*b*) Describe any methods used to examine subgroups and interactions	6
		(*c*) Explain how missing data were addressed	-
		(*d*) If applicable, explain how loss to follow-up was addressed	5
		(*e*) Describe any sensitivity analyses	-
**Results**			

For peer review only

Participants	13*	(a) Report numbers of individuals at each stage of study—eg numbers potentially eligible, examined for eligibility, confirmed eligible, included in the study, completing follow-up, and analysed	7
		(b) Give reasons for non-participation at each stage	Not applicable
		(c) Consider use of a flow diagram	18
Descriptive data	14*	(a) Give characteristics of study participants (eg demographic, clinical, social) and information on exposures and potential confounders	7
		(b) Indicate number of participants with missing data for each variable of interest	
		(c) Summarise follow-up time (eg, average and total amount)	7
Outcome data	15*	Report numbers of outcome events or summary measures over time	7
Main results	16	(*a*) Give unadjusted estimates and, if applicable, confounder-adjusted estimates and their precision (eg, 95% confidence interval). Make clear which confounders were adjusted for and why they were included	7
		(*b*) Report category boundaries when continuous variables were categorized	
		(*c*) If relevant, consider translating estimates of relative risk into absolute risk for a meaningful time period	
Other analyses	17	Report other analyses done—eg analyses of subgroups and interactions, and sensitivity analyses	
**Discussion**			
Key results	18	Summarise key results with reference to study objectives	8
**Limitations**			
Interpretation	20	Give a cautious overall interpretation of results considering objectives, limitations, multiplicity of analyses, results from similar studies, and other relevant evidence	8, 9
Generalizability	21	Discuss the generalizability (external validity) of the study results	9
**Other information**			
Funding	22	Give the source of funding and the role of the funders for the present study and, if applicable, for the original study on which the present article is based	10

*Give information separately for cases and controls in case-control studies and, if applicable, for exposed and unexposed groups in cohort and cross-sectional studies.

Note: An Explanation and Elaboration article discusses each checklist item and gives methodological background and published examples of transparent reporting. The STROBE checklist is best used in conjunction with this article (freely available on the Web sites of PLoS Medicine at http://www.plosmedicine.org/, Annals of Internal Medicine at http://www.annals.org/, and Epidemiology at http://www.epidem.com/). Information on the STROBE Initiative is available at www.strobe-statement.org.

For peer review only

BMJ Open

The magnitude and factors associated with loss to follow-up among patients treated for sexually transmitted infections: A multilevel analysis

Journal:	*BMJ Open*
Manuscript ID	bmjopen-2017-016864.R2
Article Type:	Research
Date Submitted by the Author:	12-Jun-2017

Complete List of Authors:	Tsadik, Mache; Mekelle University<br>Berhane, Yemane ; Addis Continental Institute of Public Health<br>Worku, Alemayehu; Addis Ababa University<br>Terefe, Wondwossen; Mekelle University

**Primary Subject Heading**:	Infectious diseases
Secondary Subject Heading:	Public health, Sexual health, Epidemiology
Keywords:	PUBLIC HEALTH, Infection control < INFECTIOUS DISEASES, Epidemiology < INFECTIOUS DISEASES

SCHOLARONE™
Manuscripts

For peer review only

The magnitude and factors associated with loss to follow-up among patients treated for sexually transmitted infections: A multilevel analysis

Mache Tsadik [1*,] Yemane Berhane[2], Alemayehu Worku[3], Wondwossen Terefe[1]

Mache Tsadik (adhana2008@gmail.com)

Yemane Berhane (yemaneberhane@gmail.com)

Alemayehu Worku (alemayehuwy@yahoo.com)

Wondwossen Terefe (wlerebo@uwc.ac.za)

Affiliation

1 College of health science, Mekelle University, Tigray, Ethiopia

2 Addis Continental Institute of Public Health, Addis Ababa, Ethiopia

3 Addis Ababa University, Addis Ababa, Ethiopia

* Corresponding Author, adhana2008@gmail.com

For peer review only

Abstract

Objectives: The loss to follow-up (LTFU) among patients attending care for sexually transmitted infections (STIs) in Sub-Saharan Africa is major barrier to achieving the goals of the STI prevention and control program. The objective of this study was to investigate individual- and facility-level factors associated with loss to follow-up among patients treated for STIs in Ethiopia.

Methods: A prospective cohort study was conducted among patients attending care for STIs in selected facilities from January to June 2015 in Tigray region of Ethiopia. LTFU was ascertained if a patient didn't present in person to the same facility within seven days of the initial contact. Multilevel logistic regression was used to identify factors associated with LTFU.

Results: Out of 1082 patients, 59.80% (647) were LTFU. The individual-level factors associated with LTFU included having multiple partners (AOR=2.89, 95%CI: 1.74-4.80), being male (AOR=2.23, 95%CI: 1.63-3.04), having poor knowledge about the means of STI transmission (AOR=2.08, 95%CI: 1.53-2.82), having college level education (AOR=0.38, 95%CI: 0.22-0.65) and low perceived stigma (AOR=0.60, 95% CI: 0.43-0.82). High patient flow (AOR=3.06, 95%CI: 1.30-7.18) and medium health index score (AOR=2.80, 95%CI: 1.28-6.13) were facility-level factors associated with LTFU.

Conclusions: Improving patient retention in STI follow up care requires focused interventions targeting those who are more likely to be LTFU, particularly patients with multiple partners, male index cases and patients attending facilities with high patient flow.

Key words: sexually transmitted infections, partner notification, loss to follow-up, multilevel analysis

For peer review only

Strengths and limitations of this study

- This study generated useful information that can help improve the clinical and public health interventions in the management of STIs and in reducing LTFU.
- The study used a prospective cohort design that has the potential to minimize biases related to other observational study designs.
- Advanced statistical analytic model that allow simultaneous examination of individual and facility level factors that can affect the follow up care of patients attending STI care.
- The specific reasons for LTFU were not identified because there was no established participant tracing mechanism for patients who were LTFU in the context of this study.
- The study included only STI patients attending public health facilities thus inference to patients seeking care in private facilities requires careful considerations of the local context.

For peer review only

For peer review only - http://bmjopen.bmj.com/site/about/guidelines.xhtml

Introduction:

The prevalence and incidence of sexually transmitted infections (STIs) in Sub-Saharan Africa are among the highest in the world.[1] Due to shortcomings related to laboratory capacity STI prevention and control programs adapted a syndromic management in which partner notification (PN) is a key component of treatment package.[2-4] Syndromic management is a highly sensitive approach which responds to patients symptoms and often implemented at primary health care level. As PN prevents risk of reinfection among regular partners and new infection among casual partners, treating partner(s) of the index case as early as possible is critical.[5] Thus it is important to follow index cases to assure compliance to treatment, and ascertain partner notification status [6 7] in order to effectively reduce the burden of STIs. [5 8] However, loss to follow-up (LTFU) has remained one of the challenges to effectively implement the existing treatment and preventive strategies and information on LTFU from Sub-Saharan Africa is scanty.

A number of factors have been linked to LTFU in the management of STIs cases. LTFU is more likely among males [9], single (not in union) index cases [10], individuals with low level of education compared to higher level of education [11], individuals with poor knowledge of STIs [12], and individuals who did not intend to notify partners.[13] Moreover, reluctance to return to the same facility for follow up due to fear of negative judgments.[14] On the provider side, poor quality of health services including inadequate patient education and lack of follow-up advice [15], judgmental approach of care providers, and lack of privacy and confidentiality contributes to LTFU. [10 16-18]

This study is conducted in public health facilities of Tigray region, north Ethiopia where little information is known about the magnitude of LTFU among patients treated for STIs and the associated factors. The public health facilities implement the national syndromic management protocol and treatment is provided in low charge. Therefore, the aim of this study was to investigate individual and facility-level factors associated with LTFU among STI patients attending public health facilities in North Ethiopia.

For peer review only - http://bmjopen.bmj.com/site/about/guidelines.xhtml

Methods:

The study was conducted in public health facilities of Tigray regional state, north Ethiopia. We selected health facilities with monthly patient load (STIs) of five and above in order to make the research project manageable with the resources available for the study. Thus, of the 108 public health facilities in the study area 27 fulfilled the study selection criteria. According to the national guideline, STI syndromes include vaginal discharge, urethral discharge, genital ulcer, lower abdominal pain, scrotal swelling, inguinal bubo and neonatal conjunctivitis.[19] Thus patients presenting with complaints such as burning sensation, genital discharge, genital ulcer and other related symptoms were treated as cases of STI using the Syndromic Management protocol that was adapted from the WHO generic protocol.

We conducted a prospective cohort study among patients attending public health facilities for STI care. Self-referred patients with one or more of the STI syndromes and who had sexual intercourse within three months preceding the study were recruited as study subjects. All patients that come seeking treatment for STI related symptoms during the study period were included in the study. The research team in collaboration with care providers ensured that patients receive follow-up advice and appointment. Patients were verbally consented after received routine care and informed their right to decline any time. A baseline interview was then conducted to collect relevant information from each patient by trained research nurses in a private room. Patients were informed to notify their partners and return for follow-up within 7-days. Patients received instructions and information card containing facility room where they should return and contact details of the research assistant to facilitate their follow-up visit. A minimum of two research assistants was also assigned to a health facility to capture patients returned for follow-up within the specified period.

We calculated sample size for LTFU using the assumptions of 50% LTFU by unmarried individuals, odds ratio of 1.5 at 95% CI and 80% power with non response rate of 10% which gave us a total sample size of 889. However, we had another objective aimed to determine the predictors of PN among the same population and obtained a sample size of 1095 though the

For peer review only - http://bmjopen.bmj.com/site/about/guidelines.xhtml

eligible study participants enrolled in the study were 1082. In this regard, the following assumptions were considered: 40% of married individuals who notified partners [20] and odds ratio of 1.5, 80% power with 95% CI and 20% non response rate. Thus, we took the pooled sample to increase power.

The study tool was developed by reviewing relevant literature and then adapting to the context of our study. At the individual level, the tool comprises of sociodemographic, behavioral and psychosocial components. Some of the factors considered at facility level were distance, trained providers, availability of treatment guideline, patient flow, and health index score. The tool was pre-tested before the actual use in facilities not included in the study. The study measurements were defined and described in table 1. According to the Health Management Information System (HMIS) report of Tigray Regional Health Bureau, public health facilities were classified into three levels using the health facility index score as low (<50), medium (50-74.9) and high (≥75).[21] However, among the facilities selected for this study no health facility belonged in the "low" category.

Statistical analysis:

LTFU was ascertained if the index case fails to return to the same health facility within 7-days of the initial clinic visit; and patients who were not LTFU were referred to as "in follow up care". LTFU was categorized as "Yes" for those LTFU and "No" for those retained in care.

Both individual and facility-level variables were described and presented using a simple frequency table. Before multivariate analysis was performed, Pearson's chi-square tests were used to check for the crude association between the dependent variable (LTFU) and individual and facility level variables. Then, all independent variables with *P*-value smaller than the significance level (0.05) were entered into the model.

In multilevel analyses, a null model with no covariates was used to assess the presence of significant clustering in LTFU. For individual-level factors the analysis considered sex, marital status, education, the number of partners, type of partnership, knowledge of STI transmission methods and complication, and perceived stigma. Facility-level variables include health facility index and STI patient flow. The command "xtmelogit" was used to fit a mixed-effect multilevel logistic regression model and the relationship between the dependent variable and each of the

For peer review only - http://bmjopen.bmj.com/site/about/guidelines.xhtml

independent variables (i.e. fixed effects) were assessed using odds ratios and their confidence intervals. To evaluate the significance of facility-level clustering of the dependent variable (i.e. random effects), log-likelihood ratio tests were employed. Collinearity between variables was assessed by looking at the values of variance inflation factors (VIF). VIF > 10 is assumed to be suggestive of the presence of multicollinearity. However, in this study the mean correlation value in the fitted model was 2.02.

For peer review only

Results:

A total of 1082 patients who received STI care in selected health facilities were enrolled in the study. Of which, 647 (59.80%, 95% CI: 56.88-62.72) patients who did not return for follow-up care within seven days were categorized as LTFU.

Patient and facility-level characteristics

Patient and facility level characteristics of the study sample are presented in table 2. The mean age of the cohort population was 26.4 years (SD=7.6). More than 50% of patients presented with STIs had at least high school level education. A substantial number of patients (41.22%) reported casual partnership. The majority of the health facilities (73.38%) were located in the urban settings. About three-fourth of patients reported residing within one-hour walking distance from the health facility they visited. The large proportion of LTFU (73.2%) was observed in health facilities with the high patient flow.

***Multilevel logistic regression analysis*:**

After controlling the potential confounders at individual and facility-level, the odds of LTFU were greater among index cases with multiple partners (AOR=2.89, 95%CI: 1.74-4.80), males (AOR=2.23, 95%CI: 1.63, 3.04), individuals with poor knowledge of STI transmission (AOR=2.08, 95%CI: 1.53-2.82) and poor knowledge of STI complication (AOR=1.56, 95% CI: 1.15-2.12). LTFU was less likely among better educated individuals (AOR=0.38, 95%CI: 0.22-0.65) and those with perceived low stigma (AOR=0.60, 95%CI: 0.43-0.82). LTFU was more

For peer review only - http://bmjopen.bmj.com/site/about/guidelines.xhtml

likely among patients who received care in facilities with high patient flow were three times (AOR=3.06, 95%CI: 1.30-7.18) and among patients that received care in facilities with medium health index score compared to highest index score (AOR=2.80, 95%CI: 1.28-6.13) (Table 3).

Discussion:

About two-third (59.8%) of STI patients were LTFU in this study. The individual level factors associated with increased likelihood of LTFU were being male, having multiple sexual partnerships and having poor knowledge about the means of STI transmission and their complications. While those who achieved a higher level of education and reported low perceived stigma were less likely to be LTFU. The odds of LTFU were greater among patients seen in health facility with medium health index score and in facilities with high patient flow.

The level of LTFU among STI patients is similar to other studies.[10 18] Early response to treatment within a week period and fear of stigma on return to follow-up were identified as factors contributing to LTFU.[22 23] Our study clearly indicates the potential for reinfection is quite high and that may in turn facilitate the development of drug resistance STI. In addition, since a substantial proportion of cases reported multiple sexual partnerships those untraceable and probably re-infected would continue spreading STIs in the community. This high proportion of LTFU is a major challenge to the STI prevention and control efforts and need to be addressed urgently.

The study identified a number of independent LTFU predictors both in individual and facility-level factors. In this study, males were more likely to be LTFU compared to females. This finding was consistent with studies conducted in Uganda, [24] Malawi [25] and South Africa. [26] As suggested by Geng et al., males are more likely to use substances that potentially decreases their adherence to follow-up care.[27] Males also report high risky sexual behaviors that may potentially attribute to LTFU because of linked stigma. [28]

For peer review only - http://bmjopen.bmj.com/site/about/guidelines.xhtml

The less likelihood of LTFU among educated individuals in this study is consistent with a previous study.[10]. [29],[30] This may suggest that education is an important factor in adhering to medical care.[31] Besides, highly educated individuals have a higher knowledge of STIs and individuals with high knowledge of STIs are less likely to be LTFU [12] may be because of fear of the subsequent complications. The motivation to attend follow-up care among educated individuals may be associated with their understanding of potential benefits.

LTFU was less likely among patients with low perceived stigma compared to those reported perceived high stigmas. This may show that individuals with low perceived stigma are confident enough to notify partners and have the courage to return for follow-up. [32] The stigma linked to STIs reduces the motive and willingness of index cases to notify partners and resulted in greater LTFU. [33] Fear of provider's judgmental reactions during follow-up care and embracement negatively affects follow-up considerably in low income settings.[34]

LTFU is higher among patients from facilities with a high patient flow in the present study. A similar observation was reported previously in HIV treatment setting where high patient load was associated with high proportion of LTFU.[35] The high patient load might limit the provider's time to provide adequate care that potentially influences index cases to LTFU.[36] The odds of LTFU were also lower among patients attending facilities with high index score in our study. Similarly, patient focused study conducted in Nigeria has shown that patients who received high quality care were less likely to LTFU.[37] This may indicate that high quality care motivates patients to remain in follow-up care.

This study has some important limitations. First, reasons for LTFU were not documented as this study did not have the resources to establish participant tracing mechanisms. Thus, patients who decide to do their follow up care at another health facility because of STI linked stigma may have been considered as LTFU. Second, study participants who returned for follow-up after the scheduled time were not captured. Third, since the study was conducted among self-referred patients from public health facilities, extrapolation of results to all STI patients should be made with caution since the factors related to LTFU among those seeking care in private facilities may not be similar. Behavior related information was all self-reported and may have some reliability issues because of social desirability and recall bias.

For peer review only

Despite the stated limitations, this study is one of the few studies ever conducted on LTFU among patients treated for STIs in our setting and we believe the information reported is helpful to improve interventions being implemented to prevent and control STIs in contexts similar to our study. This study also employed advanced method of analysis that allows the simultaneous analysis of individual and facility-level factors.

Conclusion:

Overall, the magnitude of LTFU among patients being treated for STIs is very high in North Ethiopia. The need for standardized follow up care and cost effective tracing mechanism is important to retain and trace STI patients who are at risk of LTFU.

Acknowledgement

We would like to acknowledge Mekelle University and Addis Continental Institute of Public Health for the overall technical and material support. This research was partially funded by African Doctoral Dissertation Research Fellowship award (ADDRF) (Grant Number: ADF 015). We would also like to thank the Tigray Health Bureau and directors of the study health facilities for facilitating the conduct of this study. We are also grateful to all study participants, data collectors and supervisors without whom this work would not have been possible.

Authors' contribution

The study was designed by MT, YB, AW and WT. MT was responsible for data collection, analysis and drafting the manuscript. YB reviewed the study design and revised the manuscript. AW supervised the data collection and analysis, and revising the manuscript, and contributed to interpretation of the analysis. WT participated in the analysis and interpretation of the data, and revised the manuscript. All authors have read and approved the final manuscript.

Funding: The study is funded by Mekelle University and African Population Health Research Center (APHRC). The funders had no role in the gathering or analysis of the data and no role in the writing of the manuscript or the decision to submit for publication.

Competing interests: The authors declare that they have no competing interests.

For peer review only - http://bmjopen.bmj.com/site/about/guidelines.xhtml

Ethical Approval: Mekelle University Health Research Ethical Review Committee (HRERC) gave ethical approval. ERC 0458/2014, date approved: 01 November 2014. The study participants gave informed consent before being involved in the study.

Data sharing statement: No additional data available

Reference:

1. World Health Organization (WHO). Global Prevalence and Incidence of Selected Curable Sexually Transmitted Infections: , 2012.
2. Golden MR, Faxelid E, and Low N. Partner notification for sexually transmitted infections including HIV infection: an evidence-based assessment.New York: McGraw-Hill. *Sex transm dis.* 2008:965-84.
3. Low N, Hawkes SJ. Putting the magic in magic bullets: top three global priorities for sexually transmitted infection control. *Sex Transm Infect.* 2011;87:44-46.
4. Centers for Disease Control and Prevention. Recommendations for Partner Services Programs for HIV Infection, Syphilis, Gonorrhea, and Chlamydial Infection. *MMWR* 2008;57. (RR-9):1-92.
5. Low N, Heijne JC, Herzog SA, et al. Reinfection by untreated partners of people treated for Chlamydia trachomatis and Neisseria gonorrhoeae: mathematical modelling study. *Sex Transm Infect* 2014;90(3):254-6.
6. Azariah S. Partner notification for sexually transmitted infections. Why can't we talk about it? *Journal of the New Zealand Medical Association* 2012;125(1355).
7. Lazaro N. Sexually Transmitted Infections in Primary Care 2013 (RCGP/BASHH). available at www.rcgp.org and www.bashh.org/guidelines, 2013.
8. Bell G, Potterat J. Partner notification for sexually transmitted infections in the modern world: a practitioner perspective on challenges and opportunities. *Sex Transm Infect. 2011; 87:34-36 doi:10.1136/sextrans-2011-050229* 2011.
9. Burnet Institute. Partner notification of sexually transmitted infections in New South Wales: An informed literature review., 2010.
10. Tang W, Huan X, Zhang Y et al. . Factors Associated with Loss-toFollow-Up during Behavioral Interventions and HIV Testing Cohort among Men Who Have Sex with Men in Nanjing, China. *PLoS ONE* 2015;10(1):e115691.
11. Ahmed I, Gugsa ST, Lemma S, et al. Predictors of loss to follow-up before HIV treatment initiation in Northwest Ethiopia: a case control study. *BMC Public Health* 2013;13(867).
12. Naome MM, Rugare KL, Michelle DN, et al. Determinants of loss to follow-up in patients on antiretroviral treatment, South Africa, 2004-2012: a cohort study. *BMC Health Services Research* 2015;15(1):1.

For peer review only - http://bmjopen.bmj.com/site/about/guidelines.xhtml

13. Wilson TE, Hogben M, Malka ES et al. A randomized controlled trial for reducing risks for sexually transmitted infections through enhanced patient-based partner notification. *Am J Public Health* 2009;99(1):104-10.
14. Ware NC WM, Geng EH, Kaaya SF, Agbaji OO, Muyindike WR, et al. Toward an understanding of disengagement from HIV treatment and care in Sub-saharan Africa: A qualitative study. *PLOS medicine* 2013;10(1):e1001369.
15. Wu H, Yim, C., Chan, A., Ho, M., and Heathcote, J. Sociocultural factors that potentially affect the institution of prevention and treatment strategies for prevention of hepatitis B in Chinese Canadians. *Can J Gastroenterol* 2009;23: 31-36.
16. Malik AI, Huppert JS. Interval to treatment of sexually transmitted infections in adolescent females. *J Pediatr Adolesc Gynecol* 2007;20(5):275-79.
17. Huppert J, Reed JL, Munafo JK, et al. Improving Notification of Sexually Transmitted Infections: A Quality Improvement Project and Planned Experiment. *PEDIATRICS* 2012;130(2):415-22.
18. Buchsbaum A, Gallo MF, Whiteman MK,. Sexually Transmitted Disease Partner Notification among African-American, Adolescent Women. *Infectious Diseases in Obstetrics and Gynecology* 2014;(2014).
19. FMOH. Syndromic managment of sexually transmited infections 2015.
20. Alam N, Streatfield PK, Khan SI, et al. Factors associated with partner referral among patients with sexually transmitted infections in Bangladesh. *Soc Sci Med* 2010;71(11):1921-6.
21. Tigray Regional Health Bureau (TRHB). Measuring index score of health facility in Tigray regional state, 2015.
22. Pickering JM, Whitworth J A G, Hughes P, et al. Aetiology of sexually transmitted infections and response to syndromic treatment in southwest Uganda. *Sex Transm Infect* 2005;81:488-93.
23. CDC. Sexually Transmitted Diseases Treatment Guidelines, 2015.
24. Amuron B, Namara G, Birungi J et al. Nabiryo C, Levin J, Grosskurth H, Coutinho A, Jaffar S. Mortality and loss-to-follow-up during the pre-treatment period in an antiretroviral therapy programme under normal health service conditions in Uganda. *BioMed Central* 2009;9:290.
25. Zachariaha R T-SK, Manzi M et al. Massaquoi M, Mwagomba B, Griensven J, Engelgem I, Arnould L, Schouten EJ, Chimbwandira FM, Harries AD. Retention and attrition during the preparation phase and after start of antiretroviral treatment in Thyolo, Malawi, and Kibera, Kenya: implications for programmes? . *Trans R Soc Trop Med Hyg* 2011;4191:10.
26. Cornell M, Grimsrud A, Fairall L, Fox MP, van Cutsem G, et al. Temporal changes in programme outcomes among adult patients initiating antiretroviral therapy across South Africa, 2002–2007. *Aids* 2010;24: 2263-2270.
27. Geng EH, Bangsberg DR, Musinguzi N, et al. Understanding reasons for and outcomes of patients lost to follow-up in antiretroviral therapy programs in Africa through a sampling-based approach. *J Acquir Immune Defic Syndr* 2010;53(3):405-11.
28. Stephenson R. Community-Level Gender Equity and Extramarital Sexual Risk-Taking Among Married Men in Eight African Countries. *International Perspectives on Sexual and Reproductive Health* 2010;36(4):178-88.
29. Panditrao M, Darak S, Kulkarni V, Kulkarni S, Parchure R. Socio-demographic factors associated with loss to follow-up of HIV-infected women attending a private sector PMTCT program in Maharashtra, India. *AIDS Care* 2011;23(5):593-600.

For peer review only - http://bmjopen.bmj.com/site/about/guidelines.xhtml

30. Ruan Y, Jia Y, Zhang X, Liang H, Li Q, et al. Incidence of HIV-1, syphilis, hepatitis B, and hepatitis C virus infections and predictors associated with retention in a 12-month follow-up study among men who have sex with men in Beijing, China. . *Journal of Acquired Immune Deficiency Syndromes* 2009;52: 604-610.
31. Tucker JS, Burnam MA, Sherbourne CD, et al. Substance use and mental health correlates of non-adherence to antiretroviral medications n a sample of patients with human immune deficiency virus infection. *Am J Med* 2003;114(7):573-80.
32. Megerso A, Garoma S, Eticha T, et al. Predictors of loss to follow-up in antiretroviral treatment for adult patients in the Oromia region, Ethiopia. *HIV/AI DS - Research and Palliative Care* 2016:8.
33. Morris JL, Lippman SA, Philip S, et al. Sexually Transmitted Infection Related Stigma and Shame Among African American Male Youth: Implications for Testing Practices, Partner Notification, and Treatment. *AIDS Patient Care STDS* 2014;28(9):499-506.
34. Bwirire LD, Fitzgerald M, Zachariah R, et al. Reasons for loss to follow-up among mothers registered in a prevention-of-mother-to-child transmission program in rural Malawi. *Trans R Soc Trop Med Hyg* 2008;102(12):1195-200.
35. Brinkhof MW, Dabis F, Myer L, et al. Early loss of HIV-infected patients on potent antiretroviral therapy programmes in low-income countries. *Bulletin of World Health Organization* 2008;86:559-67.
36. Van Damme W, Kober K, Kegels G. Scaling-up antiretroviral treatment in Southern African countries with human resource shortage: How will health systems adapt? . *Social science & medicine* 2008;66(10):2108-21.
37. Ojikutu B, Higgins-Biddle M, Greeson D, et al. The Association between Quality of HIV Care, Loss to Follow-Up and Mortality in Pediatric and Adolescent Patients Receiving Antiretroviral Therapy in Nigeria. *PLoS ONE* 2014;9(7).

For peer review only

Table-1: Description and measurements of variables in the models, North Ethiopia, 2015

Variable	***Description***	***Measurement***
Type of partnership	The relationship of index case with sexual partner (*Regular*: if there is ongoing relationship for ≥3 months. (*Casual*: if the relationship is <3 months).	Regular, Casual
STI transmission	Patient's knowledge of STI transmission: *Mean score(SD)= 2.74(1.69), 5 items*<br>1. Unprotected sexual intercourse 4. Blood transfusion<br>2. Mother to child during birth 5.Breast feeding<br>3. Injury by sharp materials (needle, blade)<br>** Response category (yes, no, I don't know) (Cronbach's Alpha =0.77)*	≥Mean=Good; <Mean=poor
STI symptoms	Patient's knowledge of STI symptoms: *Mean score(SD) = 4.03(2.04), 6 items*<br>1. Vaginal discharge 4.Pain on passing urine<br>2. Itchiness in genitalia 5.Ulcers in the gentalia<br>3. Pain/swelling in the groin 6.Eye discharge in newborn<br>** Response category (yes, no, I don't know) (Cronbach's Alpha=0.84)*	≥Mean=Good; <Mean=poor
STI prevention	Patient's knowledge of STI prevention: M*ean score(SD*) =4.62(1.00), 5 items<br>1. Abstinence 4. Use of condom<br>2. Having single partner 5. Early treatment<br>3. Avoid sex with risk partners<br>** Response category (yes, no, I don't know) (Cronbach's Alpha =0.81)*	≥Mean=Good; <Mean=poor
STI Complication	Patient's knowledge of STI complication: *Mean score(SD) =2.01(1.84), 5 items*<br>1. Caner of cervix 4. Infertility<br>2. Still birth 5. Ectopic pregnancy<br>3. Abortion<br>** Response category (yes, no, I don't know) (Cronbach's Alpha =0.82)*	≥Mean=Good; <Mean=poor
Perceived stigma	Patient's perceived stigma to PN: *Mean score(SD) = 12.92(2.52), 4 items*<br>1. Referring a partner for STI diagnosis and treatment is shame	≥Mean=high ; <Mean=low

For peer review only - http://bmjopen.bmj.com/site/about/guidelines.xhtml

	2. Attending health facility for STI treatment is embarrassed<br>3. A good man/women go to health facility for STI treatment<br>4. A good man/women notify his/her partner<br>** Response category (Very likely, likely, unlikely, very unlikely)*<br>*(Cronbach's Alpha =0.73)*	
HFI*	Health facility index:<br>It is a ranking and prioritization of health services. It is also an aggregate score of health facility performance such as availability of adequate resource, implementation plan, client satisfaction, community service etc.	High(>75),<br>Medium (50-74.9),<br>Low (<50)
HFS*	Health facility setting	Urban, Rural
Distance***	Walking distance of HF from home	≤1hrs walk,<br>>1hrs walk
STI trained**	Availability of trained care provider in STIs	Yes, No
STI patient flow*	Annual STI pt flow to health facility: *Mean score(SD) =166.26(85)*	≥Mean=high ;<br><Mean=low
Guideline**	Availability of guidelines	Yes, No

*Health management information system (HMIS) = Data from Regional Health Bureau

**Facility assessment

***patients interview

HFI=According to HMIS report, none of the selected health facility had "low" category.

For peer review only

Table-2: Profile of study subjects, North Ethiopia, 2015

Characteristic	*Loss to follow-up*		*Pearsons Chi-square (p-value)*
	Yes (%)	*No (%)*	
Gender			0.001
Female	304 (50.7)	295 (49.3)	
Male	343 (71.0)	140 (29.0)	
Age			0.930
<25 yrs	326 (59.9)	218 (40.1)	
>=25 yrs	321 (59.6)	217 (40.4)	
Education			0.001
Illiterate	140 (73.6)	50 (26.4)	
Primary	197 (60.8)	127 (39.2)	
Secondary	219 (55.1)	178 (44.9)	
College +	91 (53.2)	80 (46.8)	
Marital status			0.152
Married	264 (51.2)	252(48.8)	
Single	383 (67.7)	183(32.3)	
Residence			0.510
Urban	486 (60.4)	319 (39.6)	
Rural	161 (58.1)	116 (41.9)	
Type of partnership			0.001
Regular	346 (54.4)	290 (45.6)	
Casual	301 (67.5)	145 (32.5)	

For peer review only - http://bmjopen.bmj.com/site/about/guidelines.xhtml

For peer review only

No partners last 3 months			0.001
One	533 (56.8)	405 (43.2)	
Two or more	114 (79.2)	30 (20.8)	
Perceived stigma to PN			0.003
High	248 (63.6)	142 (39.4)	
Low	399 (57.6)	293 (42.4)	
Types of STI syndromes			0.069
Vaginal discharge	233 (44.21)	294 (55.79)	
Urethral discharge	128 (34.69)	241(65.31)	
Genital ulcer	32 (40.51)	47(59.49)	
Lower abdominal pain	28 (41.79)	39 (58.21)	
Others *	14 (35.00)	26 (65)	
Distance from health facility			0.443
<1hr walk	450 (54.6)	374 (45.4)	
≥1hr walk	197(76.4)	61 (23.6)	
Health facility index score			0.001
High	176 (50.8)	170 (49.2)	
Medium	471(64.0)	265 (36.0)	
Health facility setting			0.554
Urban	479 (60.3)	315 (39.7)	
Rural	168 (58.3)	120 (41.7)	
Patient flow to health facility			0.001
Low	324 (48.5)	344 (51.5)	
High	111(26.8)	303 (73.2)	

P value < 0.05 was considered statistically significant

For peer review only

For peer review only - http://bmjopen.bmj.com/site/about/guidelines.xhtml

Table-3: Multivariable Multilevel logistic regression analysis of individual and health facility level factors associated with LTFU North Ethiopia, 2015.

Characteristics	*Category*	*AOR (95%CI)*	*P-value*
Individual level variables			
Sex of index case			0.001
	Female	1	
	Male	2.23(1.63-3.04)	
Educational status			0.001
	Illiterate	1	
	Primary	1.11(0.71-1.74)	0.633
	Secondary	0.89(0.57-1.38)	0.613
	College +	0.38(0.22- 0.65)	0.001
Number of partner last 3months			0.001
	One	1	
	Two or more	2.89(1.74-4.80)	
Knowledge of STI transmission			0.001
	Good	1	
	Poor	2.08(1.53-2.82)	
Knowledge of STI complication			0.004
	Good	1	
	Poor	1.56(1.15-2.12)	
Perceived stigma			0.002

For peer review only - http://bmjopen.bmj.com/site/about/guidelines.xhtml

	High	1	
	Low	0.60(0.43-0.82)	
Health Facility level variables			
Health facility index			0.010
	High	1	
	Medium	2.80(1.28-6.13)	
STI patient flow			0.010
	Low	1	
	High	3.06(1.30-7.18)	
Variance		0.74*	
ICC (%)		18.56	
PCV (%)		27.88	
Model fitness			
Log likelihood		-577.92	
AIC		1155.84	

AOR=Adjusted odds ratio; ICC=Intra class correlation coefficient; 1=reference

*PCV=Proportional change in variance, AIC=Akaike information criterion * p value<0.05*

For peer review only - http://bmjopen.bmj.com/site/about/guidelines.xhtml

STROBE 2007 (v4) Statement—Checklist of items that should be included in reports of *cohort studies*

Section/Topic	Item #	Recommendation	Reported on page #
**Title and abstract**	1	(*a*) Indicate the study's design with a commonly used term in the title or the abstract	1,2
		(*b*) Provide in the abstract an informative and balanced summary of what was done and what was found	2
**Introduction**			
Background/rationale	2	Explain the scientific background and rationale for the investigation being reported	4
Objectives	3	State specific objectives, including any prespecified hypotheses	4
**Methods**			
Study design	4	Present key elements of study design early in the paper	5
Setting	5	Describe the setting, locations, and relevant dates, including periods of recruitment, exposure, follow-up, and data collection	5
Participants	6	(*a*) Give the eligibility criteria, and the sources and methods of selection of participants. Describe methods of follow-up	5
		(*b*) For matched studies, give matching criteria and number of exposed and unexposed	=
Variables	7	Clearly define all outcomes, exposures, predictors, potential confounders, and effect modifiers. Give diagnostic criteria, if applicable	5, 6,13
Data sources/ measurement	8*	For each variable of interest, give sources of data and details of methods of assessment (measurement). Describe comparability of assessment methods if there is more than one group	*13*
Bias	9	Describe any efforts to address potential sources of bias	5
Study size	10	Explain how the study size was arrived at	5
Quantitative variables	11	Explain how quantitative variables were handled in the analyses. If applicable, describe which groupings were chosen and why	6
Statistical methods	12	(*a*) Describe all statistical methods, including those used to control for confounding	6
		(*b*) Describe any methods used to examine subgroups and interactions	6
		(*c*) Explain how missing data were addressed	-
		(*d*) If applicable, explain how loss to follow-up was addressed	5
		(*e*) Describe any sensitivity analyses	-
**Results**			

For peer review only

Participants	13*	(a) Report numbers of individuals at each stage of study—eg numbers potentially eligible, examined for eligibility, confirmed eligible, included in the study, completing follow-up, and analysed	7
		(b) Give reasons for non-participation at each stage	Not applicable
		(c) Consider use of a flow diagram	18
Descriptive data	14*	(a) Give characteristics of study participants (eg demographic, clinical, social) and information on exposures and potential confounders	7
		(b) Indicate number of participants with missing data for each variable of interest	
		(c) Summarise follow-up time (eg, average and total amount)	7
Outcome data	15*	Report numbers of outcome events or summary measures over time	7
Main results	16	(*a*) Give unadjusted estimates and, if applicable, confounder-adjusted estimates and their precision (eg, 95% confidence interval). Make clear which confounders were adjusted for and why they were included	7
		(*b*) Report category boundaries when continuous variables were categorized	
		(*c*) If relevant, consider translating estimates of relative risk into absolute risk for a meaningful time period	
Other analyses	17	Report other analyses done—eg analyses of subgroups and interactions, and sensitivity analyses	
**Discussion**			
Key results	18	Summarise key results with reference to study objectives	8
**Limitations**			
Interpretation	20	Give a cautious overall interpretation of results considering objectives, limitations, multiplicity of analyses, results from similar studies, and other relevant evidence	8, 9
Generalizability	21	Discuss the generalizability (external validity) of the study results	9
**Other information**			
Funding	22	Give the source of funding and the role of the funders for the present study and, if applicable, for the original study on which the present article is based	10

*Give information separately for cases and controls in case-control studies and, if applicable, for exposed and unexposed groups in cohort and cross-sectional studies.

Note: An Explanation and Elaboration article discusses each checklist item and gives methodological background and published examples of transparent reporting. The STROBE checklist is best used in conjunction with this article (freely available on the Web sites of PLoS Medicine at http://www.plosmedicine.org/, Annals of Internal Medicine at http://www.annals.org/, and Epidemiology at http://www.epidem.com/). Information on the STROBE Initiative is available at www.strobe-statement.org.

For peer review only
